# Three-dimensional topological defects and quasi-long-range order in biological liquid crystals

**DOI:** 10.1101/2025.04.14.648711

**Published:** 2025-05-10

**Authors:** Anna E. Argento, Maria L. Varela, Gurveer Singh, Daiana P. Visnuk, Binyamin Jacobovitz, Mary E. Rutherford, Marta B. Edwards, Quentin Chaboche, Daniel A. Orringer, Jason A. Heth, Maria G. Castro, Daniel A. Beller, Carles Blanch-Mercader, Pedro R. Lowenstein

**Affiliations:** 1 Dept. of Biomedical Engineering, University of Michigan, Ann Arbor, USA.; 2 Dept. of Neurosurgery, University of Michigan Medical School, Ann Arbor, USA.; 3 Microscopy Core, Biomedical Research Core Facilities, University of Michigan, Ann Arbor, USA.; 4 Laboratoire PCC, UMR168, Institut Curie, PSL, CNRS, Sorbonne Université, 75248 Paris, France.; 5 Department of Neurosurgery, NYU Langone Health, New York, NY, USA.; 6 Dept. of Cell and Developmental Biology, University of Michigan, Ann Arbor, USA.; 7 Dept. of Physics and Astronomy, Johns Hopkins University, Baltimore, USA.

## Abstract

Active nematic liquid crystals are the main structural phase of gliomas, promoting collective migration and aggression. We establish the existence of nematic order and topological defect lines and loops in 3D in vivo mouse and human glioma brain tumors. As predicted by theory, sections through the disclination lines in 3D appear as ±1/2 topological defects in 2D. In 3D, these defects either persist along disclination lines or twist as they interconvert from −1/2 to +1/2. Cell alignment exhibits quasi–long-range order, spreading throughout the tumor over distances between 300–3000 *μ*m. In vitro −1/2 and +1/2 defects display changes in apoptosis levels, suggesting topological defects regulate glioma cell density. The large scale order of gliomas correlates with tumors’ aggressive behavior. The organization of gliomas as active nematic liquid crystals provides a novel physical foundation of complex solid tumors; their deconstruction signposts potential treatments for deadly cancers.

Whether gliomas consist of random accumulations of cells or are self-organizing remains unknown. If large scale order exists, it should manifest as invariant structures across different tumors. Active liquid crystals form a class of soft materials in which the underlying units have anisotropic shapes, typically rod-like, and form highly aligned configurations, yet also exhibit singular configurations like disclinations (1, 2, 3, 4). As active matter, the unit cells take in energy and convert it to forces, resulting in bulk, collective motion and emergent behavior. In recent works, it has been found that certain cell types in 2D in vitro, including epithelial, neuroepithelial, fibroblast, myoblasts and outer epithelium of *Hydra*, display nematic alignment and topological defects, characteristic of crystalline order (5, 6, 7, 8, 9, 10, 11). It has been proposed that crystalline order influences tissue morphogenesis, cellular extrusion and apoptosis, and mechanotransduction (5, 11, 12, 13, 14, 15, 16). These results have been obtained in 2D geometries and 2D surfaces that can deform in 3D. Work on the invasive front of breast cancer, mostly in 2D, recently suggested that nematic alignment is present within cancer cells and topological defects are present within the surrounding extracellular matrix (ECM) (17). Whether topological structures exist within tumor bulk in a 3D in vivo pathophysiological context remains to be established.

In this work, we show that glioma brain tumors in vivo, and in vitro, are structured as active nematic liquid crystals. Building on our previous work that gliomas exhibit self-organized, aligned, multicellular structures, termed oncostreams (18), we demonstrate that gliomas display nematic order, topological defects, disclinations, and quasi-long range order in 2D and in 3D. Significantly, the amount of nematic order scales with tumor aggression - suggesting crystalline order contributes to tumor malignancy - constituting a novel potential therapeutic target for this incurable cancer.

## From small- to large-scale nematic spatial organization in gliomas

**Figure 1:**
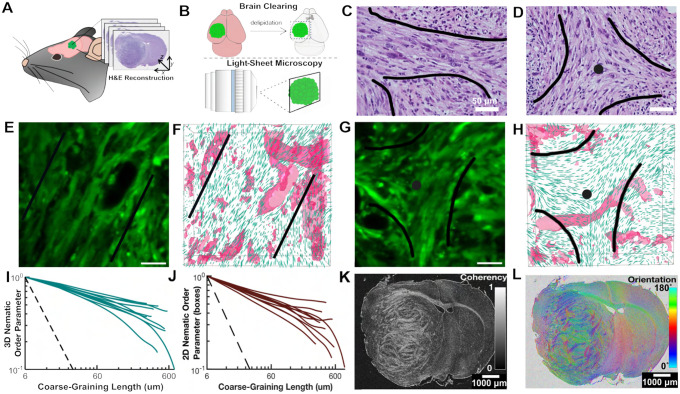
2D and 3D nematic order and topological defects in whole mouse gliomas. (**A**) Schematic of hematoxylin and eosin (H&E) 3D reconstructions of mouse brain and tumor from microtome sectioned serial 5 μm slices of paraffin embedded tissue (see Materials and Methods for more details). (**B**) Schematic of brain clearing, delipidation, tumor dissection, and 3D light sheet microscopy (LSM) imaging of GFP-tagged cleared fixed whole brains from glioma-bearing mice (see Materials and Methods for more details). (**C**) H&E stain of a region of high nematic order (oncostream) shown in 2D (inside black lines) from the mouse glioma reconstructed in panel A. (**D**) H&E stained section of the mouse glioma in panel A containing a topological defect (black lines roughly outline cell orientation and singularity marked with a dot). (**E**) Oncostream region (inside black lines) imaged with LSM from a cleared fixed whole mouse glioma. (**F**) 3D director field (green ellipsoids) corresponding to panel E. Blood vessels are outlined as pink surfaces. (**G**) Topological defect (black lines outline cell orientation and singularity marked with a dot) imaged with LSM. (**H**) 3D director field (green ellipsoids) corresponding to panel G. Blood vessels are outlined as pink surfaces. (**I**) 3D nematic order parameter against coarse-graining length for the NPD tumor sample in panels E-H. Each teal curve refers to a different ROI from the sample. (**J**) 2D nematic order parameter against coarse-graining length for the NPD tumor sample in panels E-H. Each red curve refers to a different ROI from the sample. (**K**) Coherency map of a full mouse brain and tumor H&E section (see Materials and Methods for the description of coherency). Color bar corresponds to the magnitude of the coherency. (**L**) Orientation angle color map of brain in (K). Color bar corresponds to the orientation angle. Scale bars: C, D, E, F – 50 *μ*m. K, L – 1000 *μ*m.

Gliomas display hallmarks of nematic phases: mesoscopic nematic alignment, topological defects, and quasi-long range order (QLRO). To study the large-scale 3D spatial organization in brain tumors in vivo, we reconstructed 3D sequential sections of hematoxylin and eosin (H&E) stained mouse and human tumors (Fig. 1A) and optically-cleared mouse tumors imaged directly in 3D with light-sheet microscopy (LSM) (Fig. 1B). In 2D geometries, like an H&E section of glioma-bearing mouse brains or an optical plane of cleared glioma-bearing mouse brains, mesoscopic nematic alignment was previously found in oncostreams as cells aligned along a common orientation with back-and-forth motion ((18), and Fig. 1, C and E). In nematic liquid crystals, the local direction of alignment is described by the director field (19) and topological defects are singular configurations of the director field with a non-vanishing winding number. We detected numerous examples of such defects in 2D geometries as comets (+1/2 winding number) or trefoils (−1/2 winding number) (19). Cellular arrangements that resemble trefoil defects were mainly found at the intersection between two or more oncostreams (Fig. 1, D and G).

To integrate the third dimension of gliomas, we determined the director field from high-resolution 3D imaging data of high-grade mouse gliomas (Fig. 1B, see Materials and Methods). Two cross-sections of the director field (green ellipsoids) are shown in Fig. 1, F and H, corresponding to the imaging planes in Fig. 1, E and G, respectively. To assess the degree of nematic order, we computed the 3D nematic order parameter, *S*_3*d*_, in cubic domains of varying size (coarse-graining length). This order parameter is zero for a purely random configuration and one for a perfectly aligned configuration (see Materials and Methods). We found that *S*_3*d*_ follows a power law decay, which is characteristic of QLRO, over domain sizes above 300 *μ*m (Fig. 1I). The fitted exponent was −0.29 ± 0.06 (mean±std, *n* = 10). This shows that the large-scale spatial organization of gliomas features 3D nematic alignment.

A projection of the 3D nematic alignment onto a plane illustrates both 2D nematic alignment, oncostreams (18), (Fig. 1, C and E), and configurations that resemble +1/2 and −1/2 topological defects (Fig. 1, D and G). Furthermore, the 2D nematic order parameter, *S*_2*d*_, qualitatively reproduced the behavior of *S*_3*d*_ as a function of the coarse-graining length (Fig. 1J). Importantly, the fitted parameters of the power law were comparable (−0.36 ± 0.1 vs. −0.29 ± 0.06 (*n* = 10)). This suggests that the 2D nematic order faithfully represents the 3D nematic alignment.

Moving to larger lengthscales on the order of millimeters, we found that tumor regions display a significantly larger nematic order than in normal surrounding brain regions (i.e., striatum and neocortex) in H&E samples (Fig. 1K). Although nematic order varied between tumor areas, large areas of absent order were not encountered, suggesting that tumors are mostly ordered. Interestingly, the *corpus callosum*, the bundle of axons connecting both hemispheres, displayed a high nematic order, likely the result of the parallel orientation of the axon bundles and oligodendrocytes. The further existence of crystalline order and topological defects in the *corpus callosum* was not studied further. Additionally, in tumor regions we found nematic domains of sizes from hundreds of microns to millimeters (Fig. 1L).

## Quasi-long-range nematic order in gliomas

To further characterize the large-scale nematic organization of gliomas, we analyzed the nematic order parameter for various sub-types of mouse and human tumors and brain regions (see Materials and Methods). From 3D reconstructions of sequential H&E sections, various tumor regions of interest (ROI) and normal brain ROI were analyzed. A table of all brains imaged and full H&E images are shown in fig. S1E. The high-grade mouse glioma models - NPD and NPA - and human gliosarcomas are known to be more aggressive compared to the low-grade mouse glioma model, NPAI (tumor model genetic specifications found in Materials and Methods, and survival curves shown in fig. S2) (18, 20, 21, 22). Additionally, we analyzed optically-cleared NPD mouse tumors imaged with LSM to obtain high resolution in all three dimensions (see Materials and Methods, Movie S1).

**Figure 2:**
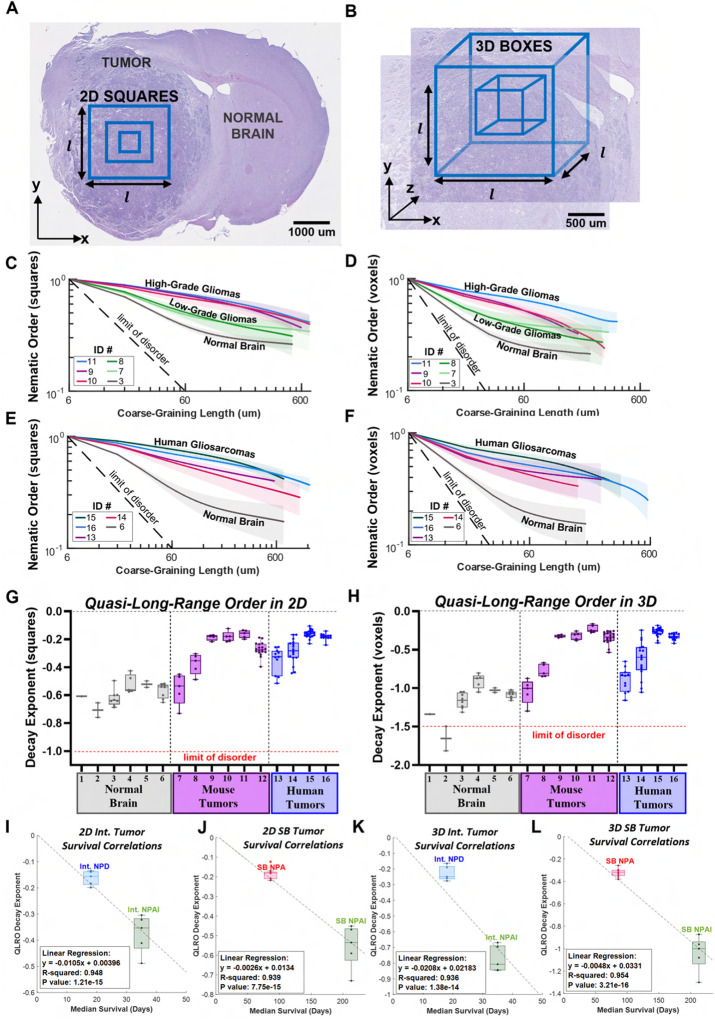
2D and 3D quasi-long range order (QLRO) in in vivo mouse and human gliomas. (**A**) An example H&E section of mouse glioma NPD tumor. The blue 2D squares represent regions of interest (ROI) for the computation of the 2D nematic order parameter (NOP) (see Materials and Methods for definition). The parameter *l* represents the coarse-graining length and corresponds to increasing sizes of the squares, scale bar = 1000 μm. (**B**) An example ROI in the H&E section of the NPD tumor in A. The blue 3D boxes represent regions for the computation of 3D NOP, scale bar = 500 μm. (**C-D**) Respectively, 2D and 3D NOPs (see Materials and Methods for definitions) as a function of the coarse-graining length (see panels A, B). The dashed line indicates the theoretical limit of a random disordered case. Colored curves show NOP for various mouse gliomas, as indicated in the legend (ID numbers found in bottom table of fig. S1). Red, blue, and purple curves are high-grade gliomas; light and dark green curves are low-grade gliomas; and gray curve is normal brain. (**E-F**) Respectively, 2D and 3D NOPs (see Materials and Methods for definitions) as a function of the coarse-graining length. The dashed line is the theoretical limit of a random case. Red, blue, green, and purple curves show NOP for various human gliosarcomas, indicated in the legend, and the gray curve shows NOP for tumor-adjacent normal brain regions. (**G-H**) Respectively, 2D and 3D decay exponents for various tumor and normal brain ROI. For each ROI, the NOP as a function of the coarse-graining length was fitted with a power law: *f* (*x*) = (1 − *c*)*x*^*b*^ + *c* (see Materials and Methods for details). The decay exponent corresponds to the fitting parameter *b*. Box and whisker plot shows each ROI’s exponent with mean shown as horizontal line. X-axis labels correspond to each tissue listed in the table in fig. S1. The horizontal red lines correspond to the exponent in the theoretical limit of a random case. (**I-L**) Respectively, correlations of 2D and 3D QLRO with mouse median survival for Intracranial and Sleeping Beauty mouse models. Abbreviations for tumor ID names defined in Materials and Methods. All brain samples and corresponding ROI shown in fig. S1. In all panels, shaded areas correspond to the standard deviation over multiple ROIs.

Due to the asymmetric resolution of sequential H&E sections (sub-micrometric in *x* and *y* and 5 − 6 *μ*m in *z*), we utilize the 2D nematic order parameter *S*_2*d*_ as a proxy for determining the 3D nematic organization (see Materials and Methods). Here, we tested two averaging domains: squares (Fig. 2A, C, and E) or boxes (Fig. 2B, D, and F). Both domains were defined by a single coarse-graining length *ℓ* (Fig. 2, A and B). Note that in squares, the in-plane director field alignment within a single image plane is evaluated, whereas in boxes, alignments from surrounding image planes are also incorporated.

For a fixed coarse-graining length, the nematic order parameter *S*_2*d*_ in low-grade and high-grade mouse gliomas and human gliosarcomas is larger than the control normal brain (Fig. 2, C–F). Additionally, the tumors’ order again propagated significantly farther than in tumor-adjacent (T-A) normal gray matter brain regions of each mouse and human samples, as indicated by the higher levels of nematic order in the tumors (figs. S3–S6). Furthermore, mouse high-grade gliomas predominantly exhibited larger magnitudes of the nematic order compared to low-grade gliomas (Fig. 2, C and D).

The nematic order parameter decays monotonically with the coarse-graining length (Fig. 2, C–F). This is expected as its magnitude tends to decrease as the length of the averaging domain increases due to, for instance, de-correlation of nematic alignment or topological defects (19, 23). Importantly, all cases deviate from the theoretical limit of a random case, *S* ~ *ℓ*^−*d*/2^, where *d* is the dimensionality of the averaging region (*d* = 2 squares, and *d* = 3 boxes), see Methods, confirming nematic correlations.

To further characterize the spatial decay of nematic alignment, we fitted the nematic order parameters with a power law with a constant deviation term, *f* (*ℓ*) = (1 − *c*) * (*ℓ*/*ℓ*_*c*_)^*b*^ + *c*, see Methods. The minimal cut-off length *ℓ*_*c*_ = 6 *μ*m was fixed. This function describes two limit cases that are controlled by a crossover length *ℓ*_*m*_ ~ *ℓ*_*c*_ (*c*/(1 − *c*))^1/*b*^: for sufficiently small coarse-graining length *ℓ* ≪ *ℓ_m_*, *f* (*ℓ*) decays as a power law with a negative decay exponent *b* < 0, which is characteristic of quasi-long range order. The decay exponent is expected to be non-universal and to depend on sample properties (24, 25). For sufficiently large coarse-graining length *ℓ* ≫ *ℓ*_*m*_, the nematic order parameter is independent of the coarse-graining length, which is characteristic of long-range order. The parameter *c* quantifies the asymptotic level of order at large length scales. In the following we refer to quasi-long range order as the cases where the nematic order parameter decays with the distance in a manner compatible with a power law. Alternatively, the cases where the nematic order parameter saturates asymptotically to a constant value are referred as long-ranged order (LRO).

The fitted values of all decay exponents are summarized in Fig. 2, G and H. We found that the decay exponent is smaller (less negative) for the human gliosarcomas, mouse NPA, and mouse NPD tumors compared to mouse NPAI (*P* value < 0.0001). Further, all tumor cases have significantly smaller decay exponents compared to the normal gray matter brain regions (NPA, NPD, gliosarcoma: *P* value < 0.0001; NPAI: *P* value = 0.007, all compared to normal brain). The tumor-adjacent normal gray matter brain regions have significantly higher (more negative) decay exponents than their neighboring tumors (Fig. 2, G and H, and figs. S3–S6).

As NPAI tumors are significantly less aggressive than NPA and NPD tumors (fig. S2, (26)), we wanted to establish whether median survival correlates with the decay exponent. Using a linear regression model, we found a negative correlation between median survival and the decay exponent, indicating that a smaller decay exponent, and thus, a higher propagation of nematic order, confers increased tumor aggression (Fig. 2, I–L). These data correlate with our previous results showing that higher density of oncostreams, the locus of nematic order in gliomas, correlates with tumor malignant behavior (18).

The fitting procedure also provides an estimate of the parameter *c*, which varies from zero (no order) to one (perfectly ordered). Whereas *b* describes the existence of QLRO, *c* describes the remaining, much lower, long-range nematic order. In the control normal brain gray matter, the value of *c* was 0.2 ± 0.04 (mean±std, n=8), and tumor-adjacent normal brain regions *c* ranged from 0.2−0.5 (fig. S7). This suggests that the normal brain has a modest intrinsic nematic order. Notably, we found that in low-grade gliomas, the value of *c* was similar to tumor-adjacent brain regions, while it was nearly vanishing for most of the high-grade gliomas analyzed (fig. S7). Altogether, this data shows that the spatial propagation of nematic order in high-grade gliomas obeys a power law decay for a length scale that ranges up to a few millimeters. In conclusion, nematic order in high-grade gliomas is quasi-long range, contrasting with the low levels of long-range order exhibited in the normal brain.

## Gliomas exhibit disclination lines and loops

Another property of nematic phases in 3D geometries is the existence of disclinations - line-like singularities in the director field, around which the director rotates by 180 degrees in a plane normal to a specific direction called the rotation vector **Ω** (23, 27). To study disclinations in whole gliomas in 3D, we focused on optically-cleared mouse tumors imaged with LSM and visualized with open-ViewMin (28) (Fig. 1B, Movie S1).

**Figure 3:**
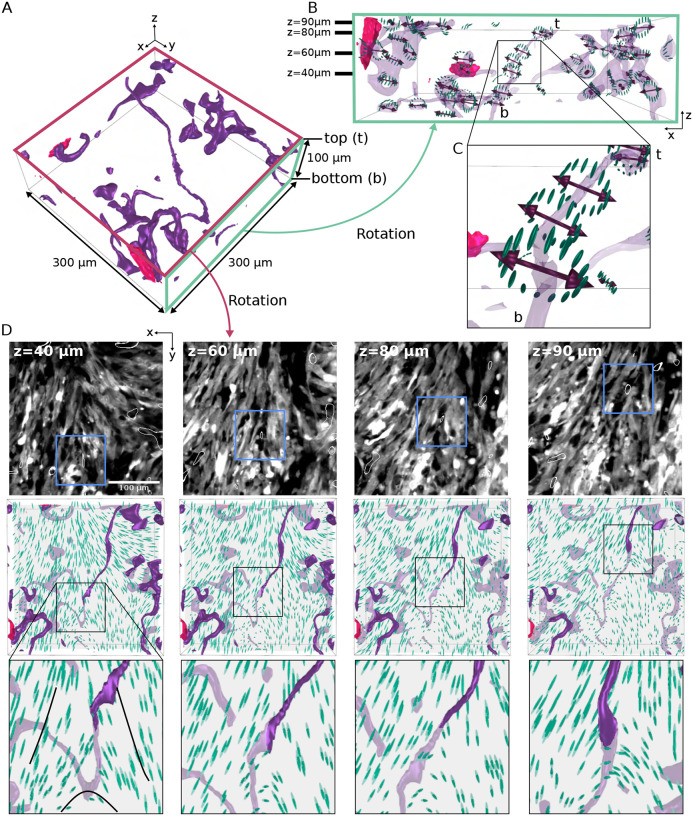
3D topological defects in in vivo mouse gliomas. (**A**) 3D reconstruction of a region (300 × 300 × 100 *μ*m^3^) from cleared intracranial NPD mouse glioma. Dark purple regions correspond to surfaces with low nematic order (see methods). Pink regions outline blood vessels. The top *z*-plane is labeled “t” and the bottom plane is labeled “b.” (**B**) Side view of the 3D reconstruction in panel A. The green rectangle corresponds to the green face in panel A. To facilitate visualization, the dark purple areas in panel A are now translucent purple. The 3D director field encircling the purple regions is shown as green ellipsoids and the double-headed red arrows represent the rotation vector, Ω. The short black lines on the top left corner of the green square label the *z*-planes that are shown in D. (**C**) Approximate zoom in of the black square in panel B. (**D**) Top row: Gray-scale LSM images of cleared tumor with blue boxes outlining disclination lines (thin white outline) through the *z*-plane (*z* coordinate indicated in top left corner). Middle row: Corresponding 3D director field (green ellipsoids) reconstructions with purple regions corresponding to surfaces with low nematic order and disclination-line locations now outlined with black boxes. Note: the *z*-plane is translucent gray, and thus the features above the *z*-plane appear in a darker color and the features below the *z*-plane in a dimmer color. Bottom row: Zoomed in view of middle row boxes showing disclination lines traveling through the *z*-plane. The scale bar is 100 *μ*m.

Surfaces bounding regions of low nematic order (purple domains in Fig. 3) have a serpentine tubular geometry. These surfaces can merge with other surfaces, split, form closed loops, or connect interfaces of vessels (pink domains in Fig. 3 and figs. S8–S10). As a result, they form an intertwined network throughout the tumor environment.

The configuration of the director field around low-nematic order surfaces reveals the existence of disclinations (Fig. 3, B and C). In some cases, the rotation vector **Ω** (double-headed arrows in Fig. 3, B and C) is nearly perpendicular to the main direction of the low-nematic order surfaces, indicating a twist profile (Movie S2). Remarkably, twist-like disclinations confirm the 3D nature of topological defects and demonstrate the necessity of the 3D nematic alignment. When scanning across *z*-planes (Fig. 3D, top panels), disclinations can be located at the intersections between two or more oncostreams, forming a trefoil-like director field configuration (Fig. 3D, middle and bottom panels). Here, the director field maintains a trefoil-like configuration along multiple z-sections of the disclination (Fig. 3D, Movies S1 and S3). However, due to the twist-like nature of this disclination, the director field configuration can resemble a comet-like topological defect, when the same disclination is viewed from another angle (fig. S10, Movie S4), further emphasizing its 3D nematic nature.

In other disclinations (figs. S8), the director configuration changed from a trefoil to a comet along the disclination line, for instance when the disclination bifurcates or forms a loop. Finally, we also observed wedge-like disclinations where the rotation vector **Ω** is nearly parallel to the main direction of the low-nematic order surfaces (fig. S9)

To further complement the above analyses, we turned to 3D reconstructions from sequential H&E sections and identified regions in high-grade tumors that appear like trefoils or comets. Numerous examples of trefoil configurations were found in NPA and NPD mouse models (figs. S11–S14). These arrangements propagated across several image planes with lengths ranging from 15 to 60 *μ*m (figs. S11–S14). With less frequency, comet-like cellular arrangements were also found in NPD mouse models with lengths between 30 to 65 *μ*m (fig. S15). Such patterns were not found within normal brain and tumor-adjacent brain sections. Altogether, our data shows that 3D disclinations are emergent structures across multiple tumor types.

## Topological defects influence cell density in gliomas

Past works have reported that several biological processes including cell extrusion, cell accumulation, and cell death can occur preferentially near topological defects (29, 30). To evaluate the potential function of topological defects, we utilized an in vitro glioma cell culture platform (fig. S16) (31). In this 2D platform, both −1/2 and +1/2 topological defects formed over 36 – 48 hours post-seeding (Movies S5–S6). Time course analysis showed that the overall nematic order parameter *S* tended to increase, while the density of topological defects tended to decrease (fig. S16). Additionally, we found that topological defect pairs of +1/2 and −1/2 can annihilate each other (fig. S16G) or remain stable (fig. S16H). Since many topological defects remain, the cell culture never reaches 100% nematic alignment (*S*_2*d*_ = 1). Finally, +1/2 defect motion was predominantly tail-to-head (fig. S17).

**Figure 4:**
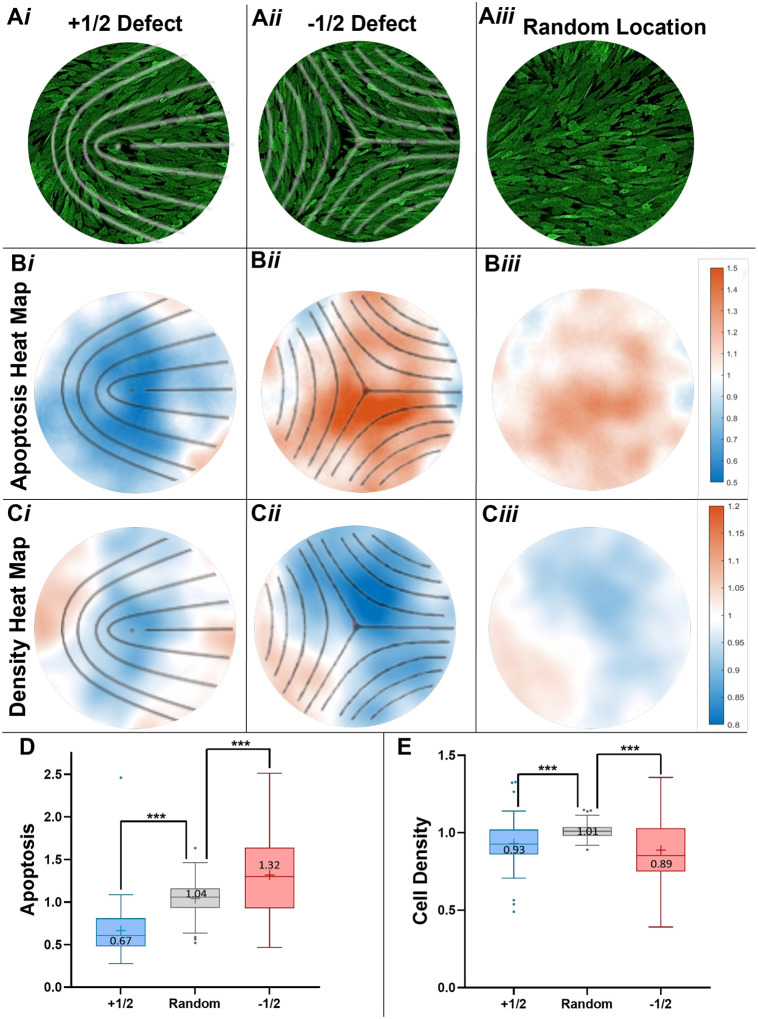
Function of topological defects in 2D glioma cells in vitro. (**A**) Confocal images of in vitro glioma cell cultures that display different nematic organization patterns: (i) +½ topological defects (overlaid in white lines), (ii) −½ topological defects (overlaid in white lines), and (iii) random location within the culture. The diameter of the circles is 224 μm. (**B**) Heat maps of average fluorescent signal of Caspase-3, indicating apoptotic cells (see Materials and Methods) for the three regions in panel A. The signals are normalized by the average of the entire field-of-view and defects are now overlaid with black lines. (**C**) Heat maps of average number of cell nuclei (stained with Hoechst, see Materials and Methods), indicating cell density for the three types of regions in panel A. The signals are normalized by the average of the entire field-of-view. (**D**) For each subpanel of B, the normalized fluorescence intensity inside a centered square region of side length 116 μm was averaged and plotted in a box and whisker plot of apoptosis (means labeled as +). (**E**) For each subpanel of C, the normalized number of cell nuclei inside a center square region of side length 116 μm was averaged and plotted in a box and whisker plot of cell density (means labeled as +). Panels D and E: n=52 for +½ defect, 45 for −½ defect, and 97 for random location with N = 3 movies. *P* values *** < 0.005.

To assess the role of topological defects in cell death, we utilized a fluorescence apoptosis marker, NucView 530 Caspase-3, in our time-lapse movies to obtain a spatiotemporal view of apoptosis (Movies S7, S8). To determine the distribution of apoptosis, we compared the apoptosis fluorescent values in randomly placed square ROIs to the same size square ROIs around topological defects (Fig. 4A). Apoptosis was drastically increased at −1/2 topological defects, by about 30%, and decreased at the head of +1/2 comets by about 33% (Fig. 4, B and D). Additionally, we determined cellular density using the nuclear dye Hoescht. −1/2 topological defects displayed an 11% lower cell density at the defect core (Fig. 4, C and E). Interestingly, cell density is also decreased by about 7% at the +1/2 defects, indicating that apoptosis and cellular density are not always inversely related and other mechanisms may decrease cell density, such as collective cell migration.

In this study, we investigated the high-order 3D organization of glioma tumors, finding that gliomas display nematic order, topological disclinations, and quasi-long range order. Additionally, increased order is positively correlated with tumor aggression. Thus, the aforementioned crystalline properties of gliomas are possible viable therapeutic targets to treat one of the most aggressive and devastating cancers with a two-year survival rate of less than 5%, as clinical strategies have remained stagnant since 2005 (21, 22, 32). Targeting the global physical order of glioma organization is an exciting and novel therapeutic approach.

## Supplementary Material

Supplement 1

1

Supplementary materials

Materials and Methods

Figs. S1 to S17

Movie S1 to S8

## Data Availability

Experimental data and the code to detect and analyze the 2D topological defects are available on Dryad (10.5281/zenodo.15199294). The code to compute the director field in 3D is available in (13).
