# Supplementary material for: Three-dimensional topological defects and quasi-long-range order in biological liquid crystals
Source: bioRxiv. 2025 May 10:2025.04.14.648711. Preprint. [Version 2] doi: 10.1101/2025.04.14.648711 (PMC12247727; doi:10.1101/2025.04.14.648711)
Supplement: 1 [file NIHPP2025.04.14.648711V2-supplement-1.pdf]

bioRxiv preprint doi: https://doi.org/10.1101/2025.04.14.648711; this version posted May 10, 2025. The copyright holder for this preprint (which was not certified by peer review) is the author/funder, who has granted bioRxiv a license to display the preprint in perpetuity. It is made available under aCC-BY 4.0 International license.

Supplementary Materials for

Three-dimensional topological defects and quasi-long-range order in biological liquid crystals

Anna E. Argento[1,2], Maria L. Varela[2], Gurveer Singh[2], Daiana P. Visnuk[2], Binyamin Jacobovitz[3], Mary E. Rutherford[2], Marta B. Edwards[2], Quentin Chaboche[4], Daniel A. Orringer[5], Jason A. Heth[2], Maria G. Castro[2,6], Daniel A. Beller[7], Carles Blanch-Mercader[4⊥], Pedro R. Lowenstein[1,2,6*]

[⊥]Corresponding author. Email: carles.blanch-mercader@curie.fr

[*]Corresponding author. Email: pedrol@umich.edu

This PDF file includes:

Materials and Methods

Figures S1 to S17

Captions for Movies S1 to S8

Other Supplementary Materials for this manuscript:

Movies S1 to S8

bioRxiv preprint doi: https://doi.org/10.1101/2025.04.14.648711; this version posted May 10, 2025. The copyright holder for this preprint (which was not certified by peer review) is the author/funder, who has granted bioRxiv a license to display the preprint in perpetuity. It is made available under aCC-BY 4.0 International license.

Materials and Methods

In Vivo Mouse Glioma Generation

Genetically Engineered Mouse Glioma Models (GEMM)

All in vivo experiments were conducted according to the guidelines approved by the Institutional Animal Care and Use Committee (IACUC) at the University of Michigan protocols PRO00011290, PRO00011291, and PRO00011292. All animals were housed in an AAALAC accredited animal facility at the University of Michigan. Animals were monitored daily. All tumor bearing animals were euthanized at the time they develop clinical signs of tumor burden. Maximal tumor burden did not exceed IACUC guidelines. Tumors harboring different genetic drivers were generated with the Sleeping Beauty (SB) transposon system, following our methodology (*20, 26*). Genetic models included the following gene expressions and inhibitions: (i) *shP5*3, *NRAS-G12V*, and *shAtrx* (NPA), (ii) *shP53*, *NRAS-G12V*, and *Pdgfβ* (NPD), (iii) *shP5*3, *NRAS-G12V*, *shAtrx*, and *IDH1-R123H* (NPAI), as in (*18, 20, 26*). Tumors were also tagged with endogenous green-fluorescent protein (GFP) for visualization. Tumors are labeled as “SB” in fig. S1E.

Intracranial Implantable Syngeneic Mouse Gliomas

Intracranial glioma tumors were generated by stereotactic intracranial implantation into the mouse striatum of $3.0{\times}10^4$ mouse glioma neurosphere cells (NPA, NPD, NPAI) in 6–8 week old females C57BL/6 mice (Taconic Biosciences) following previous methodology (*18, 20, 26*). Tumor cells are also tagged with endogenous GFP for visualization. Tumors are labeled as “Int.” in fig. S1E.

H&E tumor sections

Mice were transcardially perfused with oxygenated Tyrode’s solution, followed by 4% buffered paraformaldehyde and the brains paraffin-embedded. Brains were microtome sectioned in 5 μm thick serial sections and then stained with hematoxylin and eosin (H&E) to visualize extracellular matrix and cell cytoplasm and nuclei.

bioRxiv preprint doi: https://doi.org/10.1101/2025.04.14.648711; this version posted May 10, 2025. The copyright holder for this preprint (which was not certified by peer review) is the author/funder, who has granted bioRxiv a license to display the preprint in perpetuity. It is made available under aCC-BY 4.0 International license.

Brightfield Whole-Slide Imaging

Brightfield whole-slide scanning of serial H&E sections was performed by the University of Michigan Tissue and Molecular Pathology Shared Resource (TMPSR) Molecular Pathology Research Laboratory (MPRL) on the Vectra Polaris at 40x magnification.

Human Patient Samples

FFPE human gliosarcoma (GSC) samples were obtained from primary surgery from the University of Michigan Medical School Hospital. Patients gave informed consent for collection of tissue collection under Institutional Review Board-approved Protocol (HUM00057130) at the University of Michigan. All patients were part of the clinical trial detailed in (*33*). Samples referred to as GSC #1 and #2 are from Patient #19, GSC #3 is from Patient #20, and GSC #4 is from Patient #12 in the clinical trial. Samples were sectioned, H&E stained, and imaged as described above.

3D In Vivo Light Sheet Scanning Microscopy

Brain Clearing and Immunolabeling

To clear and label brains, the Life Canvas Active Clarity protocol was followed (*34, 35*). Mice were terminally anesthetized and perfused with oxygenated Tyrode's Solution, followed by hydrogel monomer (HM) solution containing 4% acrylamide, 0.05% bisacrylamide, 4% paraformaldehyde (PFA), 0.25% VA-044 thermal initiator in phosphate buffered saline (PBS). Brains were extracted, halved, and sectioned around the tumor, then incubated for 3-7 days at 4°C in HM solution. The brains were then purged with nitrogen gas under vacuum, then incubated overnight at 37°C. Once the solution was gelled, brains were rubbed out of the gel and incubated overnight in delipidation buffer (Life Canvas Buffer A) at 37°C. To clear lipids, brains were then placed in mesh bags, then into the Life Canvas machine with Buffer A and run for 72 hours with electrophoresis at 1000 mA. After clearing, the samples are washed with PBS at RT. To boost endogenous green fluorescence protein (GFP) signal of the glioma cells within the brain, immuno-labeling with rabbit Anti-GFP antibody (Abcam ab290) was necessary. Briefly, samples were incubated in primary sample buffer overnight, then stained for 16 hours with primary antibody and 1000 mA electrophoresis. After PBS

bioRxiv preprint doi: https://doi.org/10.1101/2025.04.14.648711; this version posted May 10, 2025. The copyright holder for this preprint (which was not certified by peer review) is the author/funder, who has granted bioRxiv a license to display the preprint in perpetuity. It is made available under aCC-BY 4.0 International license.

washes, samples were fixed with 4% PFA overnight. Samples were then incubated in secondary sample buffer and then stained for 8 hours with secondary antibody, Alexa Fluor Donkey Anti-Rabbit 488 (Fisher Scientific PIA32790), washed with secondary sample buffer alone, then PBS, and fixed overnight in 4% PFA. Index matching was performed for 24 hours in 50% EasyIndex (refractive index = 1.52) in PBS, then for 2-3 days in 100% EasyIndex. 1 µg/mL DAPI was added in the EasyIndex for nuclear staining. After refractive index matching, brains appeared translucent and barely visible within the solution.

Light Sheet Microscopy

A Zeiss Light Sheet 7 with a 20x immersion objective lens was utilized to perform broad-range *z*-stack light sheet microscopy at the University of Michigan Biomedical Research Core Microscopy Facility. The light sheet was aligned on both the right and left sides of the microscope. Large sections of tissue were imaged throughout tumor pieces approximately 1 cm^3 in size.

In Vitro 2D Experiments

Glioma Cell Lines and Culture Conditions

Mouse glioma cells were maintained at 37°C with 5% CO_2 and DMEM/F-12 media with Normocin, N2, and B27. Neurospheres were derived from our GEMMs. To produce adherent cells for in-vitro imaging, NPA neurospheres were cultured on laminin coated flasks with DMEM + 10% fetal bovine serum and used after three passages.

In Vitro Oncostream Platform

For the in vitro platform, glass coverslips or 35 mm glass-bottom culture dishes (Ibidi) were coated with Poly-D-lysine and laminin proteins as in (*31*). Culture surfaces were first acid washed with 1M hydrochloric acid for 2-4 hours at 60°C, then rinsed with milli-Q-water, and 100% ethanol. This removed dust or contamination that could possibly create artificial cell patterns. Then, poly-D-lysine at a working concentration of 100 µg/ml was applied to the culture surfaces overnight at 4°C. After drying and rinsing with sterile PBS, laminin at 100 µg/ml diluted in PBS with MgCl and CaCl was applied and incubated for 2 hours at 37°C, then at 4°C to solidify overnight. Before

bioRxiv preprint doi: https://doi.org/10.1101/2025.04.14.648711; this version posted May 10, 2025. The copyright holder for this preprint (which was not certified by peer review) is the author/funder, who has granted bioRxiv a license to display the preprint in perpetuity. It is made available under aCC-BY 4.0 International license.

cell seeding, the dish was rinsed twice with cold PBS with MgCl and CaCl and once with PBS without MgCl and CaCl. Adherent NPA cells were seeded at a density of $2x10^5$ for a 35 mm dish and incubated for 24 hours at 37°C to allow for adherence.

Apoptosis Cell Labeling

To track cellular apoptosis in time-course movies, we utilized NucView 530 Caspase3 (Biotium 10406-T) according to the manufacturer's protocols. Time lapse imaging was performed as normal with the addition of the red wavelength laser.

Time-Lapse Imaging

For time lapse imaging, we utilized an inverted Zeiss LSM880 laser scanning confocal microscope with AiryScan (Carl Zeiss, Jena, Germany) equiped with an incubation chamber set to 37°C and 5% CO_2. Cells were imaged periodically (with time interval ranging between 10-46 min), for 200-360 cycles (26 - 88 h) at 3x3 tiles at 10x or 20x resolution. We imaged 6 cultures, imaging 6-9 locations each, (n=6 movies, N=40 imaged positions).

Post-Processing of Time-Lapse Confocal Movies

After imaging cultures for up to 3 days, we utilized the FIJI plug-in OrientationJ to create vector fields of orientation for each image in the time-lapse movies. Before processing, we binned each image 2x2x1 to optimize image data size. For images taken with the 20x lens, we utilized a local window sigma of 30 px, Gaussian gradient, and 30 px grid size (25 μm). For images taken with the 10x lens, we utilized a local window sigma of 15 px, Gaussian gradient, and 15 px grid size. We then calculated the local order parameter, Q_{loc} over square sub-windows of size sigma=8 um (Eqn. 2). As there are points where alignment is disrupted, $Q_{loc} = 0$ at topological defects while $Q_{loc} = 1$ at perfectly aligned regions. We then created a local order parameter heatmap to visualize where $Q_{loc} = 0$. We found that Q_{loc} was about 1 everywhere in the images except for small, confined areas where Q_{loc} tended to 0 at the center of a topological defect. When overlaying the heatmaps onto the original images with vector fields, we found the defect locations which were corroborated visually. For the apoptosis localization analysis, the Caspase-3 channels were thresholded 24:255

bioRxiv preprint doi: https://doi.org/10.1101/2025.04.14.648711; this version posted May 10, 2025. The copyright holder for this preprint (which was not certified by peer review) is the author/funder, who has granted bioRxiv a license to display the preprint in perpetuity. It is made available under aCC-BY 4.0 International license.

on ImageJ, then Gaussian Blur 15 px was used. For spatial density analysis, the command "Make Binary" was used, and then "Gaussian Blur" set to 15 px.

Alignment of Sequential H&E Sections

Sequential H&E sections were aligned by either of the two following procedures:

1. For a given H&E section, the next H&E section was transformed by a combination of translations and rotations until optimal matching between the two sections was achieved. The criteria for optimal matching was visual inspection. This procedure was repeated until the whole series of H&E sections was aligned.

2. We used the script that is described in Ref. (*36*) to align sequential H&E sections in an automatic way. The script is available on the following GitHub page: `https://github.com/ashleylk/CODAI`. In short, this script performs a combination of transformations such as translations, rotations or interpolations to match pairs of consecutive H&E sections. Finally, the alignment of the output series of H&E sections was further improved by applying Procedure 1 on them.

The brain tumors (see fig. S1E for ID numbers): SB NPAI #1 (ID 1 and 7), Int. NPAI #2 (2 and 8), Non-Tumor Brain (3), SB NPA #1 (4 and 9), Int. NPD, (5 and 11), and SB NPA #2 (10) were aligned using Procedure 1, and the brain tumors: GSC #1 (6 and 13), GSC #2 (14), GSC #3 (15) were aligned using Procedure 2. See fig. S1 for the identity, full H&E images and ROI of the mouse and human samples.

Computational Analysis of Nematic Order and Topological Defects

Computation of the In-Plane Director Field

The computation of the in-plane director field is based on the procedure in Ref. (*37*). Below, we explain the main steps for completeness.

Let us consider a 2D intensity map $I(x, y)$, such as a fluorescence image, where (x, y) corresponds to the cartesian coordinates in two dimensions. The resolution in the two cartesian directions are considered to be equal. In this work, the resolution was set to 1 μm. Next, a Gaussian filter with

bioRxiv preprint doi: https://doi.org/10.1101/2025.04.14.648711; this version posted May 10, 2025. The copyright holder for this preprint (which was not certified by peer review) is the author/funder, who has granted bioRxiv a license to display the preprint in perpetuity. It is made available under aCC-BY 4.0 International license.

standard deviation σ_1 was used on $I(x, y)$. This step eliminated small-wavelength fluctuations in the intensity map. Finally, for every position (x, y), we computed the gradients of the intensity map $(\partial_x I, \partial_y I)$ and constructed a structure matrix M_2, which takes the form

$$M_2 = \begin{pmatrix} \langle \partial_x I * \partial_x I \rangle & \langle \partial_x I * \partial_y I \rangle \\ \langle \partial_x I * \partial_y I \rangle & \langle \partial_y I * \partial_y I \rangle \end{pmatrix} - \frac{Tr}{2}\mathbb{I} \tag{S1}$$

where $\mathbb{I}$ is the identity matrix. The variable $Tr = \partial_x I * \partial_x I + \partial_y I * \partial_y I$, and therefore the matrix M_2 is traceless. The symbol $\langle \cdot \rangle$ denotes a spatial average over a surrounding region at the position (x, y). In practice, we used a second Gaussian filter with standard deviation σ_2.

For every position (x, y), the matrix M_2 is diagonalized. Then, the eigenvector with the smallest eigenvalue was defined as the in-plane director field $\mathbf{n}_2(x, y)$. By definition, the in-plane director field $\mathbf{n}_2(x, y)$ is a unit vector (i.e. $|\mathbf{n}_2(x, y)| = 1$). Besides, by definition $\mathbf{n}_2$ and its opposite are equivalent, meaning that $\mathbf{n}_2 \rightarrow -\mathbf{n}_2$ are indistinguishable.

In conclusion, this method computes the in-plane director field $\mathbf{n}_2$ from an intensity map $I(x, y)$ and two input parameters σ_1, and σ_2. In this work, the value of $\sigma_1 = 1\ \mu$m was the same as the image resolution, and the value of $\sigma_2 = 6\ \mu$m.

Computation of the 3D Director Field

The computation of the 3D director field is based on a generalization of the procedure in Ref. (37) from two to three dimensions. Below, we explain the main steps to determine the 3D director field from a 3D intensity map.

Let us consider a 3D intensity map $I(x, y, z)$, such as a z-stack of fluorescence images, where (x, y, z) corresponds to the Cartesian coordinates in three dimensions. Besides, the resolution in the three Cartesian directions is considered to be the same. In this work, the resolution was set to $1\ \mu m$. Next, a Gaussian filter with standard deviation σ_1 was used on $I(x, y, z)$. This step eliminated small-wavelength fluctuations in the intensity map. Finally, for every position (x, y, z), we computed the gradients of the intensity map $(\partial_x I, \partial_y I, \partial_z I)$ and constructed a structure matrix M_3, which takes the form

$$M_3 = \begin{pmatrix} \langle \partial_x I * \partial_x I \rangle & \langle \partial_x I * \partial_y I \rangle & \langle \partial_x I * \partial_z I \rangle \\ \langle \partial_x I * \partial_y I \rangle & \langle \partial_y I * \partial_y I \rangle & \langle \partial_y I * \partial_z I \rangle \\ \langle \partial_x I * \partial_z I \rangle & \langle \partial_y I * \partial_z I \rangle & \langle \partial_z I * \partial_z I \rangle \end{pmatrix} - \frac{Tr}{3}\mathbb{I} \tag{S2}$$

bioRxiv preprint doi: https://doi.org/10.1101/2025.04.14.648711; this version posted May 10, 2025. The copyright holder for this preprint (which was not certified by peer review) is the author/funder, who has granted bioRxiv a license to display the preprint in perpetuity. It is made available under aCC-BY 4.0 International license.

where $\mathbb{I}$ is the identity matrix. The variable $Tr = \partial_x I * \partial_x I + \partial_y I * \partial_y I + \partial_z I * \partial_z I$, and therefore the matrix M_3 is traceless. The symbol $\langle\cdot\rangle$ denotes a spatial average over a surrounding region at the position (x, y, z). In practice, we used a second Gaussian filter with standard deviation σ_2.

For every position (x, y, z), the matrix M_3 was diagonalized. Then, the eigenvector with the smallest eigenvalue was defined as the director field $\mathbf{n}_3(x, y, z)$. By definition the director field $\mathbf{n}_3(x, y, z)$ was a unit vector (i.e. $|\mathbf{n}_3(x, y, z)| = 1$). Besides, by definition $\mathbf{n}_3$ and its opposite are equivalent, meaning that $\mathbf{n}_3 \to -\mathbf{n}_3$ are indistinguishable. Note that the $\mathbf{n}_3$ includes the orientation in the (out-of-plane) z direction, unlike to the in-plane director field $\mathbf{n}_2$ that was described in the previous section.

In conclusion, the method computes a 3D director field $\mathbf{n}_3(x, y, z)$ from an 3D intensity map $I(x, y, z)$, and two input parameter σ_1, and σ_2. In this work, the value of $\sigma_1 = 1$ μm was the same as the image resolution, and the value of $\sigma_2 = 6$ μm.

Computation of 2D Nematic Order Parameters

Below, we explain the procedure to compute from a 3D intensity field $I(x, y, z)$, two variables that are linked to the nematic order parameter and in the main text, we named 2D nematic order parameter.

For a fixed $z = z_0$ plane, we computed the in-plane director field $\mathbf{n}_2$ from the intensity map $I(x, y, z = z_0)$, using the method that was described in "Computation of the In-Plane Director Field." We used this approach because the resolution of H&E sections is lower in the z direction than in the x and y directions. The resolution in x and y was set to 1 μm. The resolution in z was approximately set to a fixed value of 6 μm because the spacing between two consecutive H&E sections was estimated to be 5 μm but roughly 5 of every 6 sections were analyzed, meaning that the average spacing between analyzed section was 6 μm. From the in-plane director field $\mathbf{n}_2(x, y, z = z_0)$, we determined the phase $\phi(x, y, z = z_0)$ as $\mathbf{n}_2(x, y, z = z_0) = (\cos(\phi(x, y, z = z_0)), \sin(\phi(x, y, z = z_0)))$, where the phase $\phi(x, y, z = z_0)$ is the angle between the in-plane director field $\mathbf{n}_2(x, y, z = z_0)$ and the x-axis. Then, the phase $\phi(x, y, z = z_0)$ was binned by a factor 6 in the x and y directions to match the resolution in x, y, z. Therefore after this transformation, a unit pixel of ϕ corresponds to 6 μm. Finally, repeating these operations for every z plane, leads to a 3D map of the phase $\phi(x, y, z)$.

bioRxiv preprint doi: https://doi.org/10.1101/2025.04.14.648711; this version posted May 10, 2025. The copyright holder for this preprint (which was not certified by peer review) is the author/funder, who has granted bioRxiv a license to display the preprint in perpetuity. It is made available under aCC-BY 4.0 International license.

Next, we computed the 2D nematic order parameter $S_{2d}(\ell)$ as

$$S_{2d}(\ell) = \sqrt{\langle \cos(2\phi) \rangle_\ell^2 + \langle \sin(2\phi) \rangle_\ell^2} \tag{S3}$$

where the symbol $\langle \cdot \rangle$ denotes a spatial average in the surrounding region of the position (x, y, z). In the main text, we computed (S3), in two types of averaging regions: a square in the $x - y$ plane and a box. In practice, for the former region, we used a 2D square filter, which was computed using the function imboxfilt from Matlab with a filterSize of ℓ. Besides, for the latter region, we used a 3D box filter, which was computed using the function imboxfilt3 from Matlab with a filterSize of ℓ. Note that the two regions are characterized by a single length scale ℓ that is named coarse-graining length. Besides, note that the 2D nematic order parameter that is computed in squares compares the alignment of the in-plane director field within a single image plane, whereas the 2D nematic order parameter that is computed in boxes includes the in-plane director field from other image planes. Finally, $S_{2d}(\ell)$ was averaged for all positions in the region of interest.

In the case of imaging data from light sheet microscopy, the resolution in x, y and z was equal and set to 1 μm. To compute the 2D nematic order parameters $S_{2d}(\ell)$, the phase ϕ was binned by a factor 6 in the three cartesian directions. After this transformation a unit pixel corresponds to 6 μm. This allowed to compared the curves of the 2D nematic order parameters with those obtained from 3D reconstructions from sequential H&E sections.

Finally, the range of coarse-graining length studied in experiments was set as follows: the minimal coarse-graining length is 6 μm, which corresponds to one pixel after binning. The maximal coarse-graining length was set by the smallest dimension of a region of interest.

This procedure was used to compute the 2D nematic order parameter shown in Fig. 2, C-F, and figs. S3-S6.

Spatial Dependence of the Nematic Order Parameter

A nematic order parameter, such as $S_{2d}(\ell)$ in "Computation of 2D Nematic Order Parameters" can present three types of scalings with the coarse-graining length ℓ. Long-ranged nematic order is when the nematic order parameter is independent of ℓ, $S(\ell) \sim constant$. Quasi-long ranged nematic order is when the nematic order parameter decays as a power law with ℓ, $S(\ell) \sim \ell^{-\alpha}$, where α is the decay exponent. Finally, short-ranged nematic order is when the nematic order parameter

bioRxiv preprint doi: https://doi.org/10.1101/2025.04.14.648711; this version posted May 10, 2025. The copyright holder for this preprint (which was not certified by peer review) is the author/funder, who has granted bioRxiv a license to display the preprint in perpetuity. It is made available under aCC-BY 4.0 International license.

decays exponentially with ℓ, $S(\ell) \sim \exp(-\ell/\lambda)$, where λ is the decay length. Although in statistical physics, these definitions apply in the limit where the coarse-graining length approaches infinity $\ell \to \infty$, here we roughly use the same definitions to describe our experimental system, which has a large cut-off scale set by the system size.

It is instructive to revisit the scaling of the nematic order parameter $S_{2d}(\ell)$ in Eq. (S3) for a random distribution of orientations. For more details, we refer to (7). Let us consider that the phase ϕ is a set of independent random variables that are uniformly distributed between 0 and π. Then, the nematic order parameter scales as $S_{2d} \sim 1/\sqrt{N}$, where N is the number of independent random variables in the surrounding region. Since in images, the density of pixels is constant, the number N is related to the coarse-graining length as $N \sim l^d$, where d is the dimension of the surrounding region ($d = 2$ for squares and $d = 3$ for boxes). Therefore, the nematic order parameter scales as $S_{2d}(\ell) \sim \ell^{-d/2}$ for a random case. The limit of the random case is shown in Fig. 2, and figs. S3- S6 as a black dashed curve.

Fitting Procedure of the Curves of 2D Nematic Order Parameter

The procedure to fit the curves of 2D nematic order parameter that were obtained by the procedure explained in "Computation of the 2D Nematic Order Parameters" is the following: For each curve of the 2D nematic order parameter, $S_{2d}(\ell)$, the power law with a deviation term $f(\ell) = (a-c)(\ell/\ell_c)^b + c$ was fitted, where ℓ corresponds to the coarse graining length, which in our case corresponds to the length of either a square or a box. The parameter $\ell_c = 6$ μm is set to the smallest coarse-graining length. The fitting parameter a was constrained in the range of 0.995 to 1.005 because by definition of the nematic order parameter $S_{2d}(\ell = 1px) = 1$. The other fitting parameter b and c were unconstrained. The exponent shown in Fig. 2 corresponds to b and the asymptotic nematic order at large length scales shown in fig. S7 corresponds to c.

Computation of the 3D Nematic Order Parameter

From the 3D director field $\mathbf{n}_3$ obtained by the method described in "Computation of the 3D Director Field," we computed the 3D nematic order parameter as follows:

1. For every position (x, y, z), compute a nematic tensor q from the component of the director

bioRxiv preprint doi: https://doi.org/10.1101/2025.04.14.648711; this version posted May 10, 2025. The copyright holder for this preprint (which was not certified by peer review) is the author/funder, who has granted bioRxiv a license to display the preprint in perpetuity. It is made available under aCC-BY 4.0 International license.

field $\mathbf{n}_3 = (n_x, n_y, n_z)$. The nematic tensor q takes the form

$$q = \begin{pmatrix} n_x^2 - Tr/3 & n_x n_y & n_x n_z \\ n_x n_y & n_y^2 - Tr/3 & n_y n_z \\ n_x n_z & n_y n_z & n_z^2 - Tr/3 \end{pmatrix} \tag{S4}$$

where $Tr = n_x^2 + n_y^2 + n_z^2 = 1$ because the director field $\mathbf{n}_3$ is a unit vector (i.e. $|\mathbf{n}_3| = 1$).

2. For every position (x, y, z), compute the coarse-grained nematic tensor $Q_3^c(\ell) = \langle q \rangle$, where the symbol $\langle \cdot \rangle$ denotes a spatial average in the surrounding region of the position (x, y, z). In practice, we used a box filter, which was computed using the function imboxfilt3 from Matlab with a filterSize of ℓ. The parameter ℓ is the coarse-graining length.

3. For every position (x, y, z), diagonalize the coarse-grained nematic tensor $Q_3^c(\ell)$. Since by definition, the nematic tensor is uniaxial, the diagonalised form of $Q_3^c(\ell)$ reads

$$Q_3^c(\ell) = S_{3d}(\ell) \begin{pmatrix} 2/3 & 0 & 0 \\ 0 & -1/3 & 0 \\ 0 & 0 & -1/3 \end{pmatrix} \tag{S5}$$

where $S_{3d}(\ell)$ is the 3D nematic order parameter.

This procedure was applied only to the case of imaging data from light sheet microscopy.

Finally, we compared the curves of 2D nematic order parameters that were computed in boxes to the 3D nematic order parameter $S_{3d}(\ell)$, Fig. 1, I and J. As it is expected the two nematic order parameters decay with the coarse-graining length ℓ, Fig. 1, I and J. Next, we fitted a power law with exponent b and a deviation term c as explained in "Fitting Procedure of the Curves of 2D Nematic Order Parameter." The average decay exponent of S_{3d} is -0.29 ± 0.06 (mean±SD, $n = 10$), was similar to the average decay exponent of S_{2d} is -0.36 ± 0.10. Besides the average values of c was also similar between the two cases: 0.09 ± 0.14 for S_{3d} and 0.08 ± 0.17 for S_{2d}. Therefore, this suggests the scaling of the 2D nematic order parameter was a good metric of the 3D nematic alignment.

bioRxiv preprint doi: https://doi.org/10.1101/2025.04.14.648711; this version posted May 10, 2025. The copyright holder for this preprint (which was not certified by peer review) is the author/funder, who has granted bioRxiv a license to display the preprint in perpetuity. It is made available under aCC-BY 4.0 International license.

Computation of Low Nematic Order Isosurfaces

The coarse-grained nematic tensor Q_3^c was computed as explained in the above Section, except that the director field $\mathbf{n}_3$ was not binned by a factor 6. The coarse graining length was set to $\ell = 31\ \mu$m.

To visualize the coarse-grained nematic tensor Q_3^c, we used the software Open-ViewMin at `https://gitlab.com/open-viewmin/open-viewmin.gitlab.io`. The green ellipsoids in Fig. 3 and figs. S8-S10 represent the director field associated with Q_3^c, which is uniaxial by definition. The purple surfaces in the same figures correspond to low nematic order isosurfaces. These were also computed using the software Open-ViewMin. In short, the surfaces are defined by the condition that the largest eigenvalue of Q_3^c is equal to a number λ. In our case, this number was between $\lambda = 0.2$ and 0.23. The white outlines in the same figures correspond to cuts of a low nematic order surface with a z plane. We checked that the shape of the isosurface and the director field were qualitatively similar upon small changes in both the parameter λ and ℓ.

Finally, in the same figures, the pink regions roughly represent vessels. These regions were determined by binarising images based on an intensity threshold that was adjusted for each region of interest independently.

Analysis of +1/2 Topological Defect Motion

Here, we analyzed time-lapse images of cultures for up to 16 h to 77 h post-confluency. For each time point, the two-dimensional director field $\mathbf{n}_{2d}$ was computed using the method explained in "Computation of the In-Plane Director Field" using a value of $\sigma_2 = 21\ \mu$m. Half-integer topological defects were identified as regions where the $2d$ nematic order parameter $S_{2d} < 0.2$ and the winding number of the director field is $\pm 1/2$. Here S_{2d} was computed using a Gaussian filter with standard deviation of 7 μm. The polarity $\mathbf{p}$ of $+1/2$ topological defects is computed using Eq. (5) in Ref. (38). If two topological defect trajectories are closer than 150 μm, both defects are eliminated to exclude motion due to defect interactions.

Tracking of $+1/2$ topological defects was performed using the Python module `trackpy` with the parameter 'search range' = 83 μm and the parameter 'memory'= 50 frames, (39). The second parameter is between 8 h and 38 h depending on the movie. If a defect trajectory consists of less than 10 frames or its length is shorter than 30 μm, it is eliminated.

bioRxiv preprint doi: https://doi.org/10.1101/2025.04.14.648711; this version posted May 10, 2025. The copyright holder for this preprint (which was not certified by peer review) is the author/funder, who has granted bioRxiv a license to display the preprint in perpetuity. It is made available under aCC-BY 4.0 International license.

Finally, we analyzed the motion of the remaining $+1/2$ topological defects, $N = 172$, see fig. S17A. For each of trajectory, we computed the angle difference $\Delta\theta$ between the mean defect polarization, and the displacement vector from the last time point to the first time point, see fig. S17B. The mean defect polarization was computed as the mean of $\mathbf{p}$ over a trajectory. The motion of the defect was classified as head-to-tail, if $-45^\circ < \Delta\theta < 45^\circ$, tail-to-head, if $225^\circ < \Delta\theta < 135^\circ$, and 'other' in any other case, see fig. S17C. The results in fig. S17D show that $+1/2$ defect motion was predominantly tail-to-head.

bioRxiv preprint doi: https://doi.org/10.1101/2025.04.14.648711; this version posted May 10, 2025. The copyright holder for this preprint (which was not certified by peer review) is the author/funder, who has granted bioRxiv a license to display the preprint in perpetuity. It is made available under aCC-BY 4.0 International license.

Figure S1: H&E brain samples from human gliosarcoma patients and mouse models.

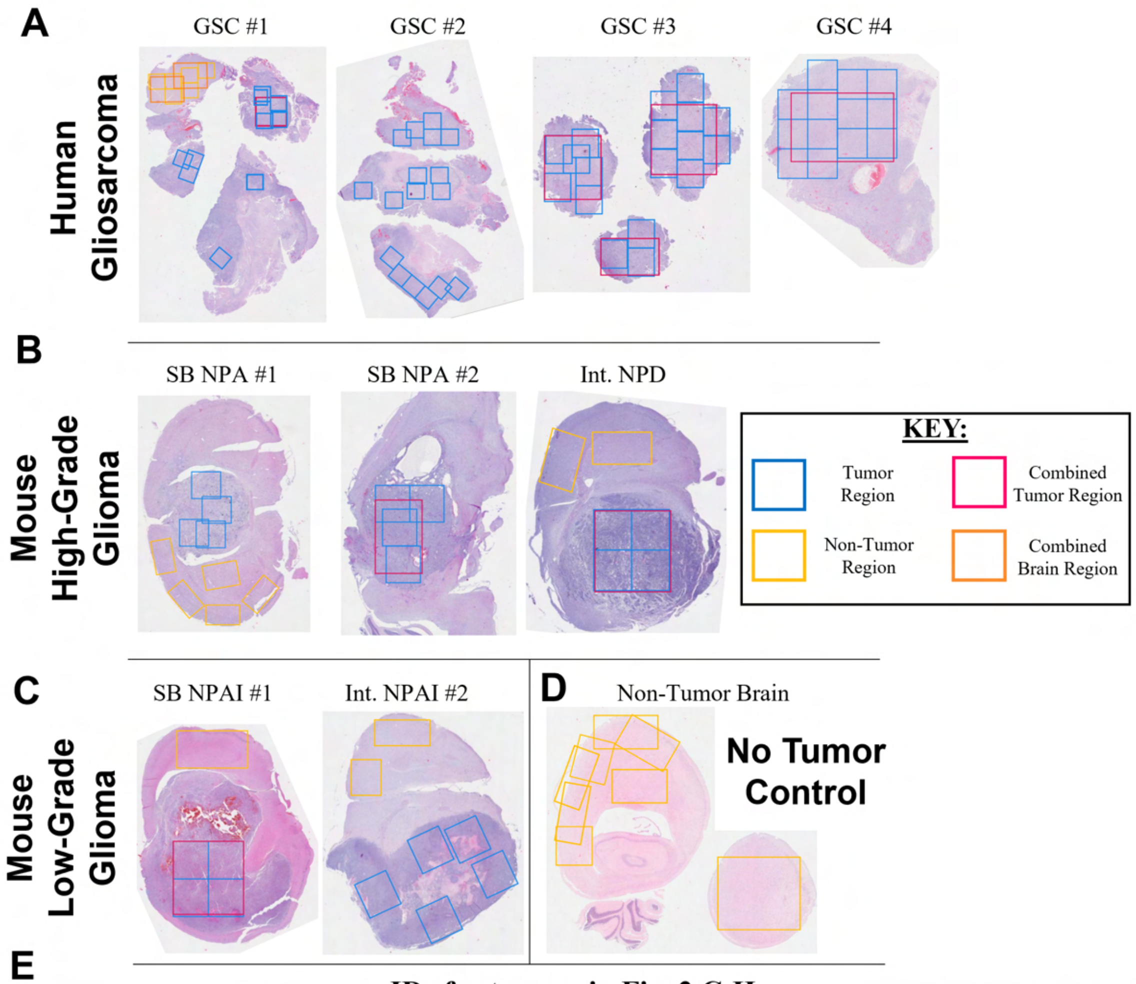

E

IDs for tumors in Fig. 2 C-H

ID	Name	Species	# ROI	ID	Name	Species	# ROI	ID	Name	Species	# ROI
1	SB NPAI #1 Brain	Mouse	1	7	SB NPAI #1 Tumor	Mouse - LGG	5	13	GSC #1 Tumor	Human - HGG	11
2	Int. NPAI #2 Brain	Mouse	2	8	Int. NPAI #2 Tumor	Mouse - LGG	5	14	GSC #2 Tumor	Human - HGG	17
3	Non-Tumor Brain	Mouse	8	9	SB NPA #1 Tumor	Mouse - HGG	4	15	GSC #3 Tumor	Human - HGG	21
4	SB NPA #1 Brain	Mouse	5	10	SB NPA #2 Tumor	Mouse - HGG	5	16	GSC #4 Tumor	Human - HGG	14
5	Int. NPD Brain	Mouse	2	11	Int. NPD Tumor	Mouse - HGG	5				
6	GSC #1 Brain	Human	8	12	Int. NPD Tumor (Cleared)	Mouse - HGG	18				

bioRxiv preprint doi: https://doi.org/10.1101/2025.04.14.648711; this version posted May 10, 2025. The copyright holder for this preprint (which was not certified by peer review) is the author/funder, who has granted bioRxiv a license to display the preprint in perpetuity. It is made available under aCC-BY 4.0 International license.

Figure S1: **H&E brain samples from human gliosarcoma patients and mouse models.** Examples of H&E sections of each brain and tumor analyzed. **(A)** Human gliosarcoma (GSC) patient samples. **(B)** High-grade mouse gliomas type NPA and NPD. **(C)** Low-grade mouse glioma models type NPAI. **(D)** Normal brain control with no tumor. Regions of interests (ROIs) are outlined with color-coded boxes to illustrate the regions analyzed. Blue are tumor regions, pink are larger combined tumor regions, yellow are non-tumor regions like tumor-adjacent normal brain regions or brain control regions, and orange are larger combined non-tumor regions. **(E)** Corresponding brain/tumor names for ID numbers found in Main Figure 2 with animal species and numbers of ROI listed. Abbreviations for tumor ID names defined in Materials and Methods. The acronym SB stands for Sleeping Beauty and the acronym Int. for intracranial tumor generation methods.

bioRxiv preprint doi: https://doi.org/10.1101/2025.04.14.648711; this version posted May 10, 2025. The copyright holder for this preprint (which was not certified by peer review) is the author/funder, who has granted bioRxiv a license to display the preprint in perpetuity. It is made available under aCC-BY 4.0 International license.

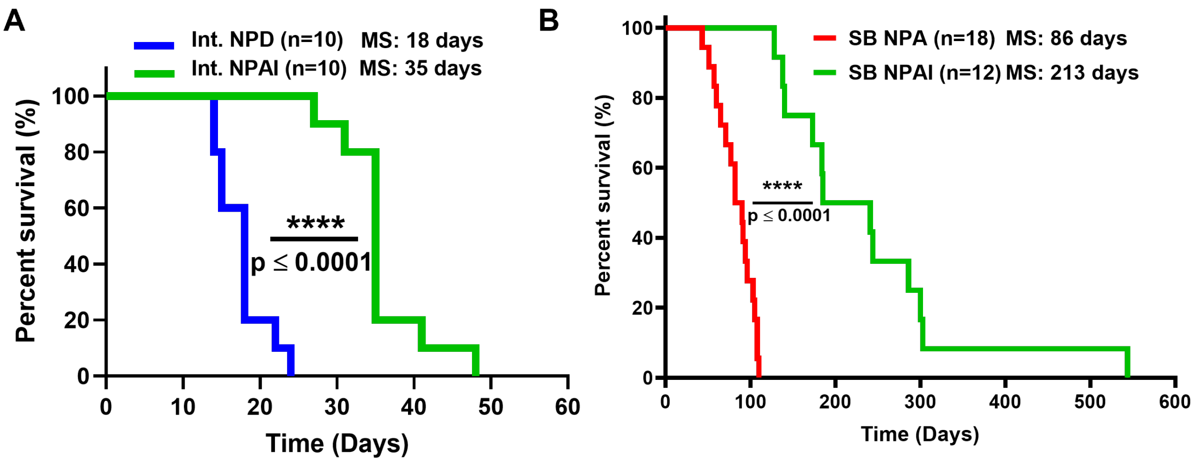

Figure S2: **Glioma Survival Curves**. **(A)** Kaplan-Meier survival curves for Int. NPD and Int. NPAI mouse tumors (*P* value $*** \leq 0.0001$). **(B)** Kaplan-Meier survival curves for SB NPA and SB NPAI mouse tumors (*P* value $*** \leq 0.0001$). Abbreviations for tumor ID names defined in Materials and Methods. NPA and NPD are high-grade gliomas while NPAI is low-grade. Figure modified from (*18*).

bioRxiv preprint doi: https://doi.org/10.1101/2025.04.14.648711; this version posted May 10, 2025. The copyright holder for this preprint (which was not certified by peer review) is the author/funder, who has granted bioRxiv a license to display the preprint in perpetuity. It is made available under aCC-BY 4.0 International license.

Figure S3: Nematic Order Parameter in Mouse Tumors in 2D.

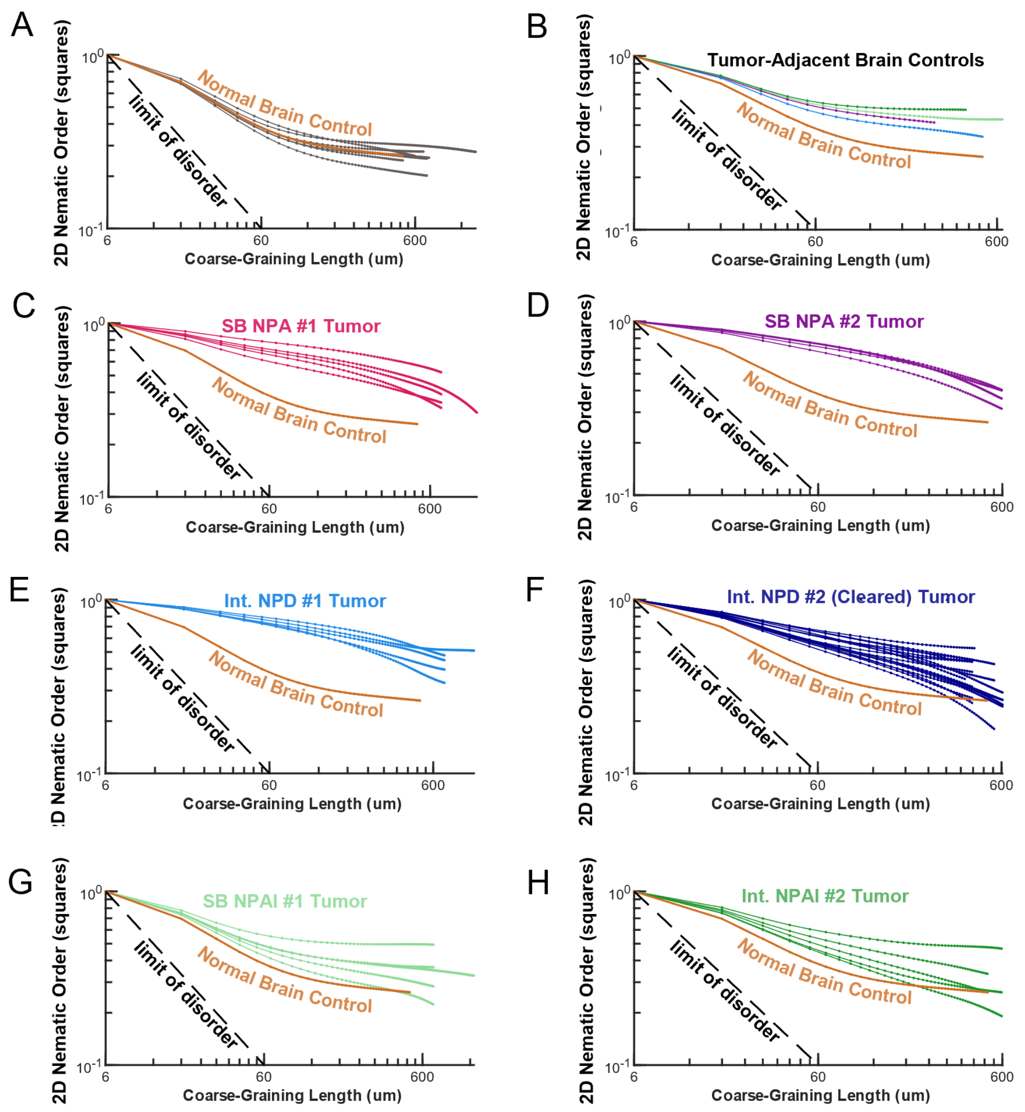

bioRxiv preprint doi: https://doi.org/10.1101/2025.04.14.648711; this version posted May 10, 2025. The copyright holder for this preprint (which was not certified by peer review) is the author/funder, who has granted bioRxiv a license to display the preprint in perpetuity. It is made available under aCC-BY 4.0 International license.

Figure S3: **Nematic Order Parameter in Mouse Tumors in 2D.** (A-H) 2D Nematic Order Parameter (NOP) as a function of coarse-graining length of the averaging domains (squares) is shown for a variety of mouse tumors of different aggression levels (C-H), paired tumor-adjacent regions (B), and a control normal brain (A). High Grade Gliomas: panels C-F. Low Grade Gliomas: panels G-H. Each tumor or normal brain piece is segmented into several smaller regions which are plotted in the same color. The average NOP of the normal brain control graphed in panel A is shown as the orange solid curve in all panels. The black dashed curve in all panels represents the theoretical limit of a random case $y = x^{-1}$. Color keys: (A) Control brain containing no tumor - gray. (B) tumor-adjacent brain controls from tumors shown in (D,E,G,H) with matching colors. (C) SB NPA #1 tumor - pink. (D) SB NPA #2 tumor - purple. (E) Int. NPD #1 tumor - light blue. (F) Int. NPD #2 tumor, imaged with clearing - dark blue. (G) SB NPAI #1 tumor - light green. (H) Int. NPAI #2 tumor - dark green. Abbreviations for tumor ID names defined in Materials and Methods. The acronym SB stands for sleeping beauty and the acronym Int. for intracranial.

bioRxiv preprint doi: https://doi.org/10.1101/2025.04.14.648711; this version posted May 10, 2025. The copyright holder for this preprint (which was not certified by peer review) is the author/funder, who has granted bioRxiv a license to display the preprint in perpetuity. It is made available under aCC-BY 4.0 International license.

Figure S4: Nematic Order Parameter in Mouse Tumors in 3D.

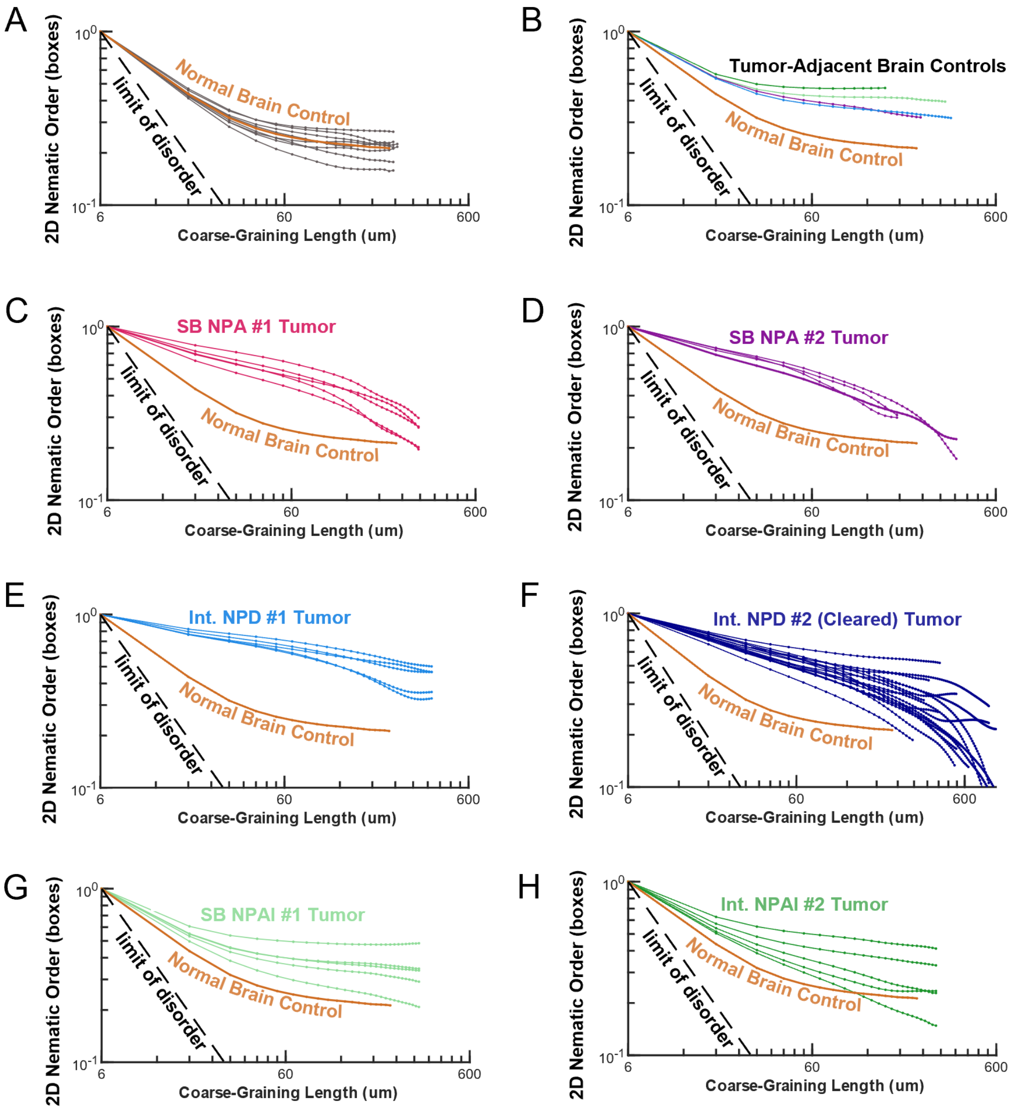

Figure S4: **Nematic Order Parameter in Mouse Tumors in 3D.** Same as Figure S3, but plotting the 2D Nematic Order Parameter (NOP) as a function of coarse-graining length of the averaging domains in **3D boxes**. Here, the theoretical limit of a random case is $y = x^{-1.5}$.

bioRxiv preprint doi: https://doi.org/10.1101/2025.04.14.648711; this version posted May 10, 2025. The copyright holder for this preprint (which was not certified by peer review) is the author/funder, who has granted bioRxiv a license to display the preprint in perpetuity. It is made available under aCC-BY 4.0 International license.

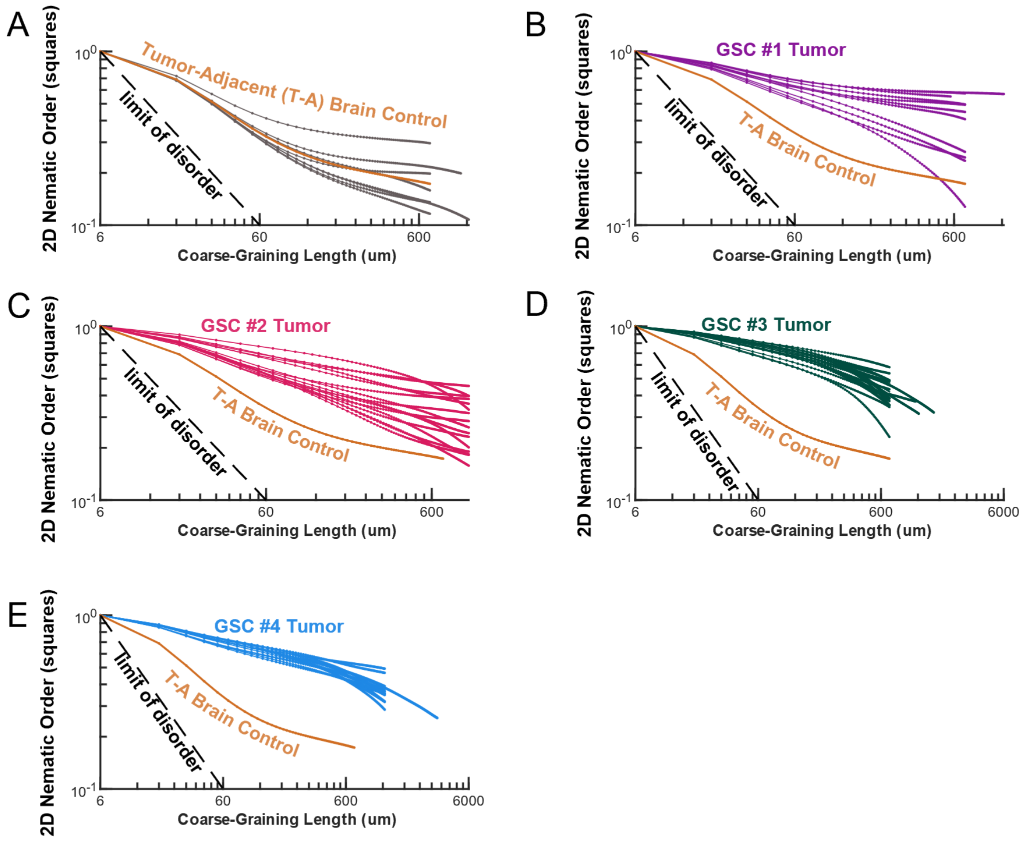

Figure S5: **Nematic Order Parameter in Human Gliosarcoma Tumors in 2D.** Same as Figure S4, but plotting for a variety of human gliosarcoma (GSC) tumors and a control tumor-adjacent normal brain region. Color keys: (A) GSC #1 tumor-adjacent normal brain control regions - gray. (B) GSC #1 tumor - purple. (C) GSC #2 tumor - pink. (D) GSC #3 tumor - dark green. (D) GSC #2 tumor - blue. Abbreviations for tumor ID names defined in Materials and Methods and S1E.

bioRxiv preprint doi: https://doi.org/10.1101/2025.04.14.648711; this version posted May 10, 2025. The copyright holder for this preprint (which was not certified by peer review) is the author/funder, who has granted bioRxiv a license to display the preprint in perpetuity. It is made available under aCC-BY 4.0 International license.

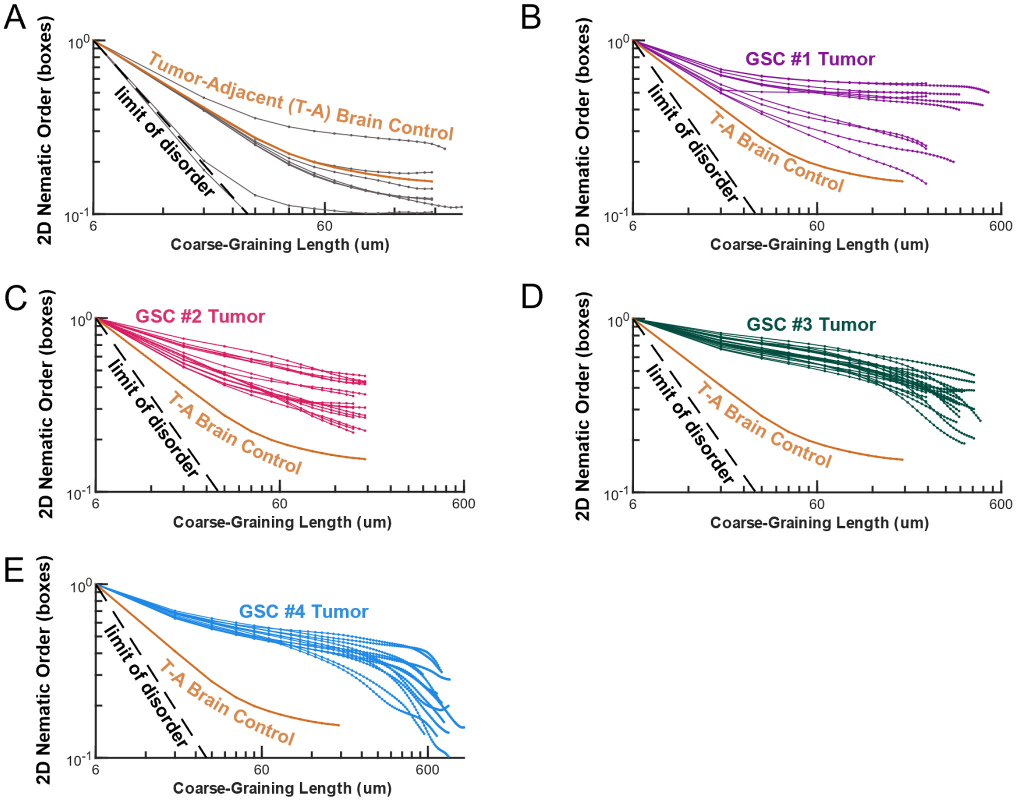

Figure S6: **Nematic Order Parameter in Human Gliosarcoma Tumors in 3D.** Same as Figures S3 and S5, but plotting the 2D Nematic Order Parameter (NOP) as a function of coarse-graining length of the averaging domains in **3D boxes** in human gliosarcoma samples. Here, the theoretical limit of a random case is $y = x^{-1.5}$.

bioRxiv preprint doi: https://doi.org/10.1101/2025.04.14.648711; this version posted May 10, 2025. The copyright holder for this preprint (which was not certified by peer review) is the author/funder, who has granted bioRxiv a license to display the preprint in perpetuity. It is made available under aCC-BY 4.0 International license.

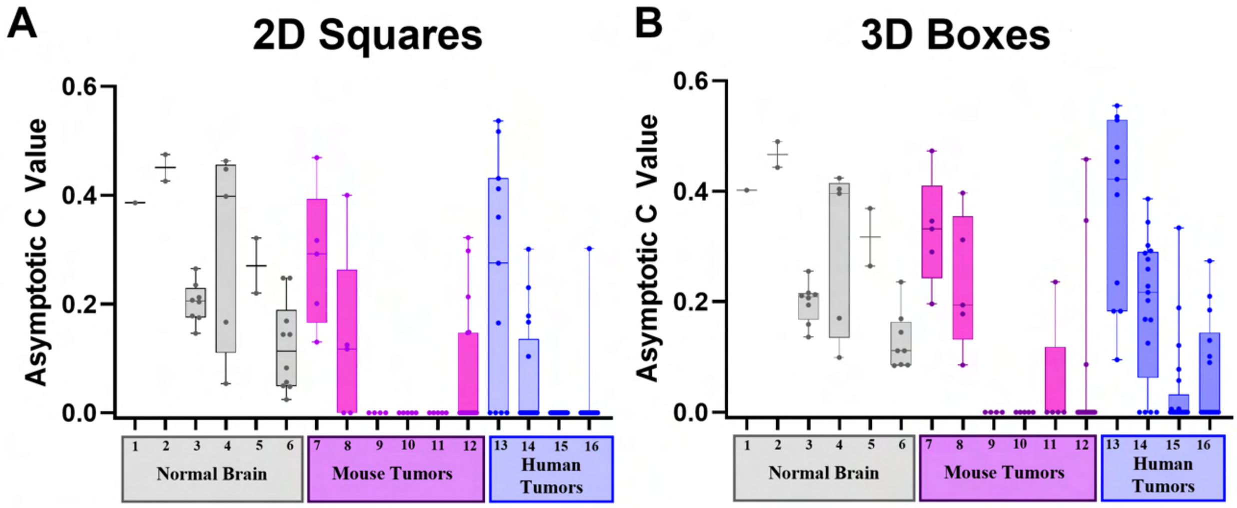

C

ID	Name	Species	# ROI	ID	Name	Species	# ROI	ID	Name	Species	# ROI
1	SB NPAI #1 Brain	Mouse	1	7	SB NPAI #1 Tumor	Mouse - LGG	5	13	GSC #1 Tumor	Human - HGG	11
2	Int. NPAI #2 Brain	Mouse	2	8	Int. NPAI #2 Tumor	Mouse - LGG	5	14	GSC #2 Tumor	Human - HGG	17
3	Non-Tumor Brain	Mouse	8	9	SB NPA #1 Tumor	Mouse - HGG	4	15	GSC #3 Tumor	Human - HGG	21
4	SB NPA #1 Brain	Mouse	5	10	SB NPA #2 Tumor	Mouse - HGG	5	16	GSC #4 Tumor	Human - HGG	14
5	Int. NPD Brain	Mouse	2	11	Int. NPD Tumor	Mouse - HGG	5				
6	GSC #1 Brain	Human	8	12	Int. NPD Tumor (Cleared)	Mouse - HGG	18				

Figure S7: **2D and 3D asymptotic level of nematic order at large length scales.** (**A-B**) Respectively, 2D and 3D asymptotic c values for various tumor and normal brain regions of interest (ROI). For each ROI, the 2D nematic order parameter curves that is computed in either squares or boxes was fitted with a power law: $f(x) = (1-c)x^b + c$ (see Materials and Methods for definitions), respectively. The asymptotic level of nematic order at large length scales corresponds to the fitting parameter c. Box and whisker plot shows each ROI's value of c with mean shown as horizontal line. X-axis labels correspond to each tissue listed in the table of panel fig. S1E, shown again in panel (**C**). Shaded areas correspond to the standard deviation over multiple ROIs.

bioRxiv preprint doi: https://doi.org/10.1101/2025.04.14.648711; this version posted May 10, 2025. The copyright holder for this preprint (which was not certified by peer review) is the author/funder, who has granted bioRxiv a license to display the preprint in perpetuity. It is made available under aCC-BY 4.0 International license.

Figure S8: Example of a twist-type disclination loop

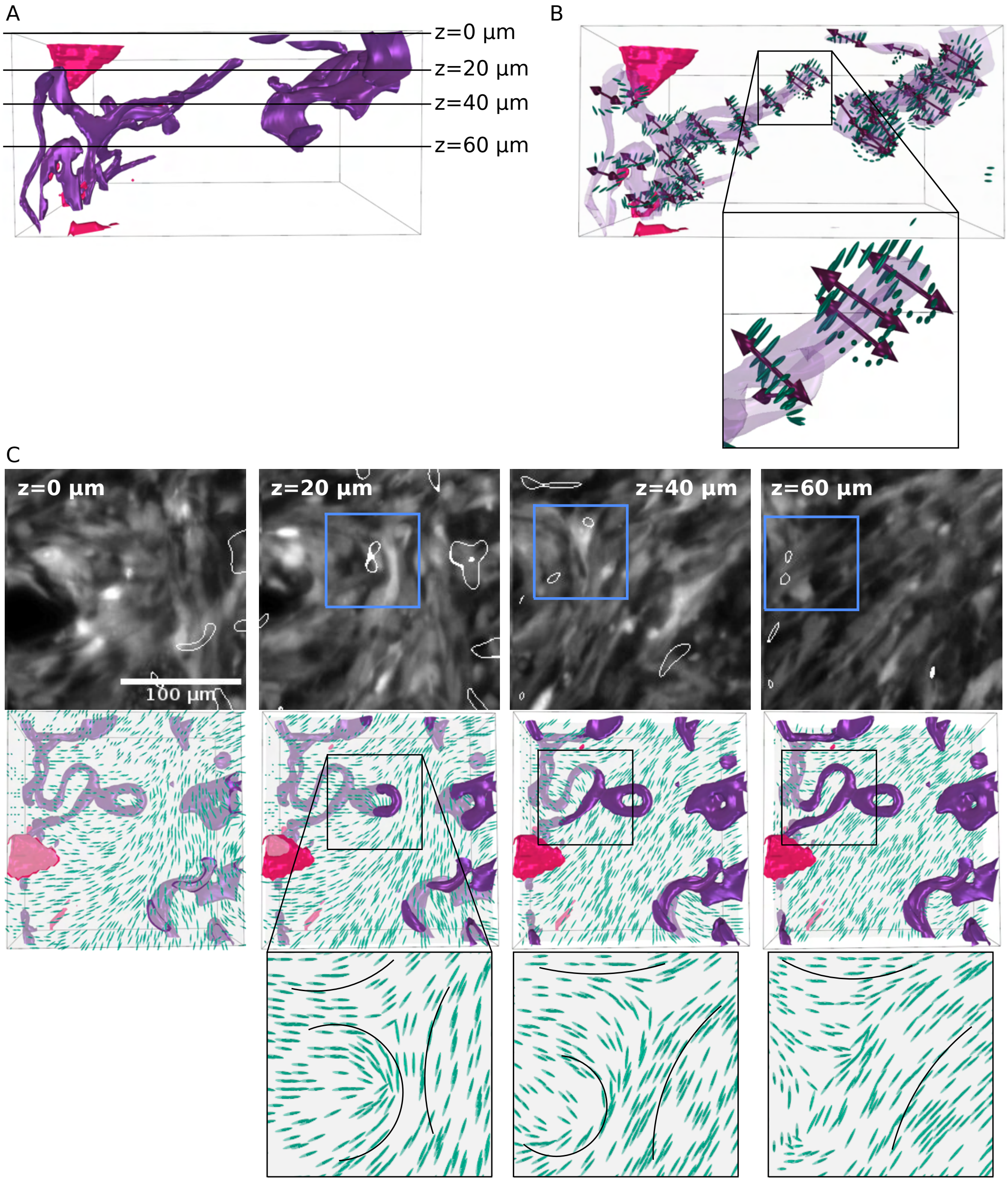

bioRxiv preprint doi: https://doi.org/10.1101/2025.04.14.648711; this version posted May 10, 2025. The copyright holder for this preprint (which was not certified by peer review) is the author/funder, who has granted bioRxiv a license to display the preprint in perpetuity. It is made available under aCC-BY 4.0 International license.

Figure S8: **Example of twist-type disclination loop (A)** 3D reconstructions of a region from cleared intracranial NPD mouse glioma. Dark purple regions correspond to surfaces with low nematic order. Pink regions outline blood vessels. The black lines label the z-planes that are shown in C. (**B**) Detailed side view of the 3D reconstruction in panel A. To facilitate visualization, the dark purple areas in panel A are now translucent purple. The 3D director field in the surrounding purple regions is shown as green ellipsoids and the double-headed red arrows represent the rotation vector, Ω. Below: approximate zoom in of the black square in panel B. (**C**) Top row: Gray-scale LSM images of cleared tumor with blue boxes outlining disclination lines (thin white outline) through the z-plane (z coordinate indicated in top left corner in white). Middle row: Corresponding 3D director field (green ellipsoids) reconstructions with purple regions corresponding to surfaces with low nematic order and disclination-line locations now outlined with black boxes. Note: the z-plane is translucent gray, and thus the features above the z-plane appear in a darker color and the features below the z-plane in a dimmer color. Bottom row: Zoomed in view of disclination lines traveling through the z-plane. In the left-most column, there is no panel as the disclination of interest does not appear at $z = 0$ μm (see panel A). The scale bar is 100 μm.

bioRxiv preprint doi: https://doi.org/10.1101/2025.04.14.648711; this version posted May 10, 2025. The copyright holder for this preprint (which was not certified by peer review) is the author/funder, who has granted bioRxiv a license to display the preprint in perpetuity. It is made available under aCC-BY 4.0 International license.

Figure S9: Example of a wedge-type disclination line that starts and ends on a blood vessel.

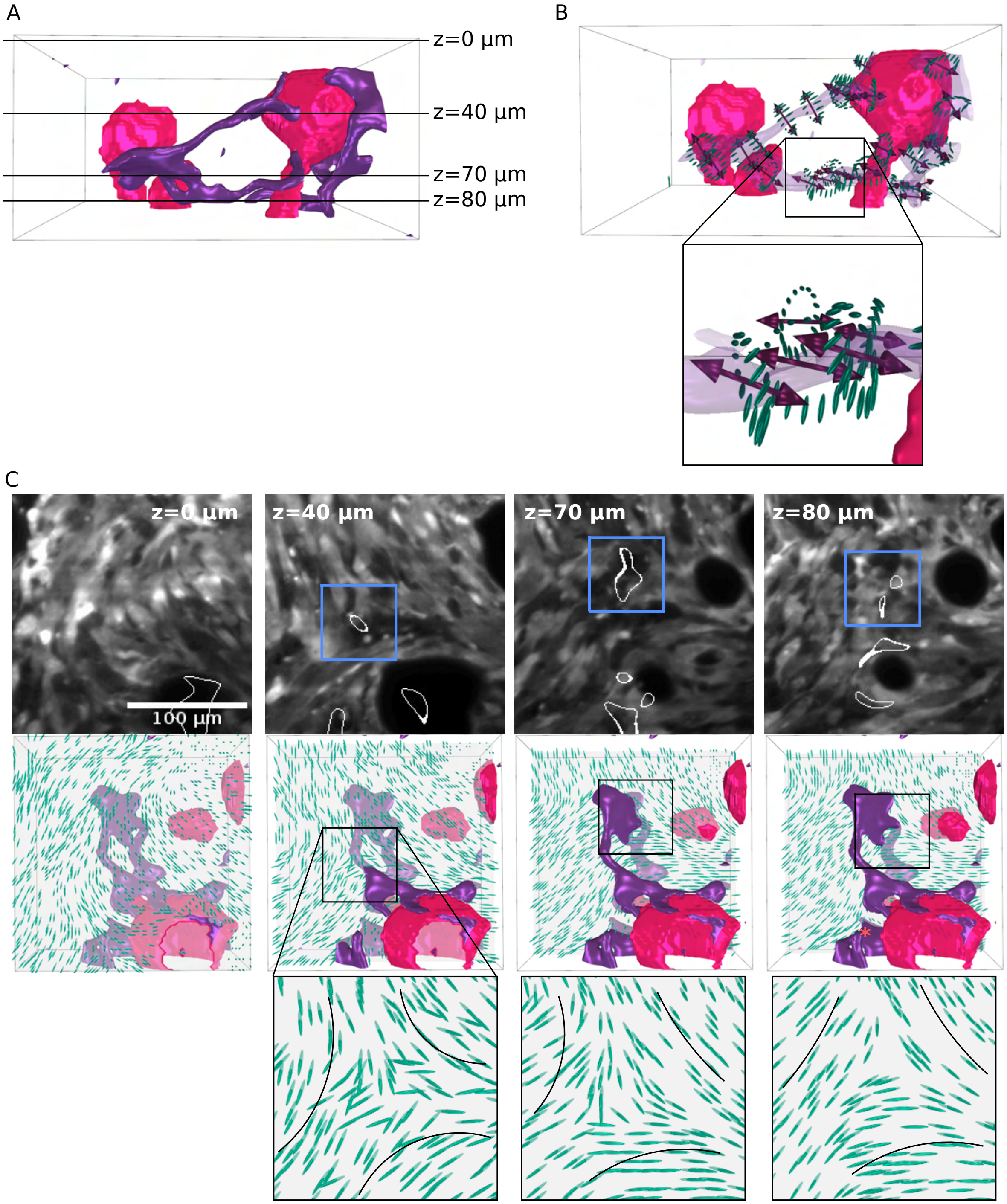

bioRxiv preprint doi: https://doi.org/10.1101/2025.04.14.648711; this version posted May 10, 2025. The copyright holder for this preprint (which was not certified by peer review) is the author/funder, who has granted bioRxiv a license to display the preprint in perpetuity. It is made available under aCC-BY 4.0 International license.

Figure S9: **Example of a wedge-type disclination line that starts and ends on a blood vessel.** (A) 3D reconstructions of a region from cleared intracranial NPD mouse glioma. Dark purple regions correspond to surfaces with low nematic order. Pink regions outline blood vessels. The black lines label the z-planes that are shown in C. (**B**) Detailed side view of the 3D reconstruction in panel A. To facilitate visualization, the dark purple areas in panel A are now translucent purple. The 3D director field in the surrounding purple regions is shown as green ellipsoids and the double-headed red arrows represent the rotation vector, $\mathbf{\Omega}$. Below: zoom in of the black square in panel B. (**C**) Top row: Gray-scale LSM images of cleared tumor with blue boxes outlining disclination lines (thin white outline) through the z-plane (z coordinate indicated in top corner of image). Middle row: Corresponding 3D director field (green ellipsoids) reconstructions with purple regions corresponding to surfaces with low nematic order and disclination-line locations now outlined with black boxes. Note: the z-plane is translucent gray, and thus the features above the z-plane appear in a darker color and the features below the z-plane in a dimmer color. Bottom row: Zoomed in view of disclination lines traveling through the z-plane. In the left-most column, there is no panel as the disclination of interest does not appear at $z = 0$ μm (see panel A). The scale bar is 100 μm.

bioRxiv preprint doi: https://doi.org/10.1101/2025.04.14.648711; this version posted May 10, 2025. The copyright holder for this preprint (which was not certified by peer review) is the author/funder, who has granted bioRxiv a license to display the preprint in perpetuity. It is made available under aCC-BY 4.0 International license.

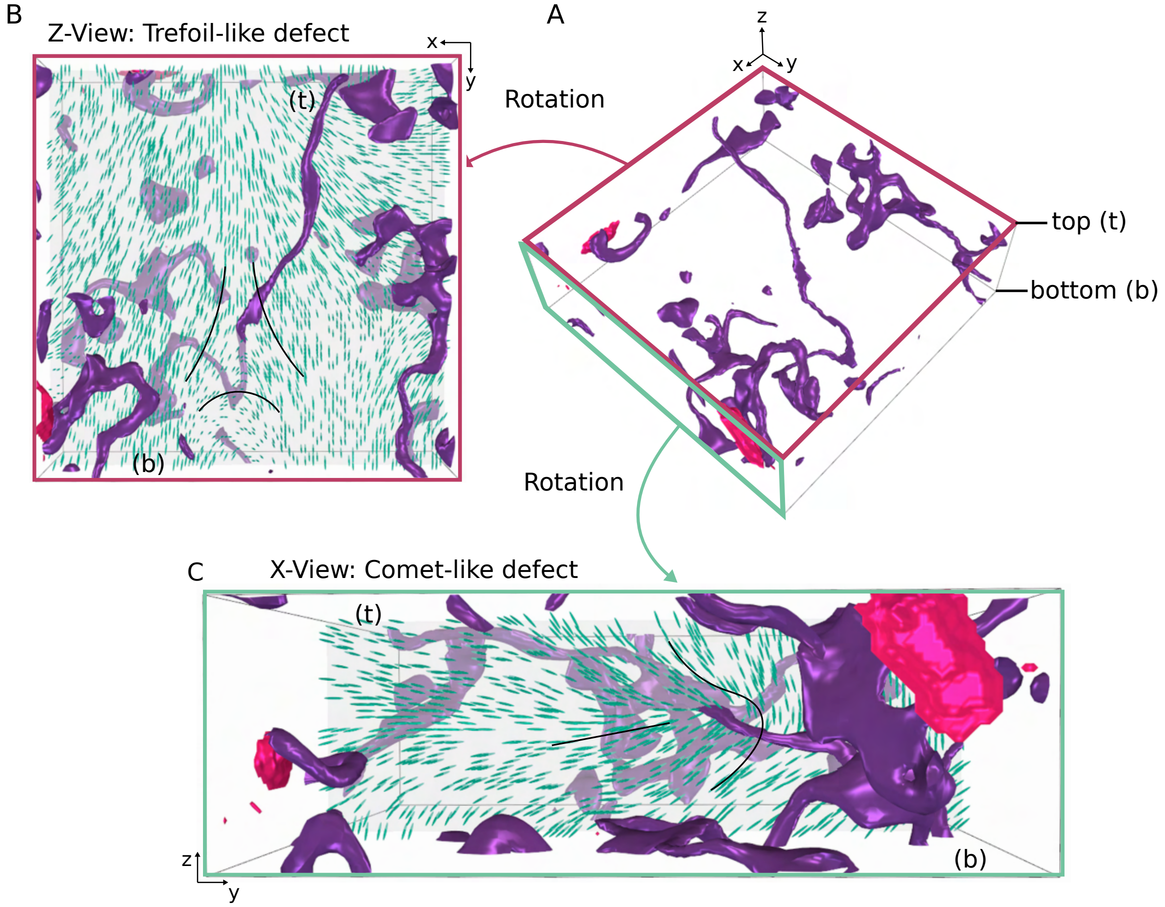

Figure S10: **Two cross-sections of a twist-like disclination.** (**A**) Same 3D reconstruction as in panel A in Fig. 3. Dark purple regions correspond to surfaces with low nematic order (see Methods). Pink regions roughly outline blood vessels. The top z-plane is labeled (t) and the bottom plane is labeled (b). (**B**) Z-view of the 3D reconstruction in panel A. The red outline corresponds to the red face in panel A. 3D director field is shown as green ellipsoids. In this view, the defect appears as a -1/2 trefoil. (**C**) X-view of the 3D reconstruction in panel A. The green outline corresponds to the green face in panel A. To improve visualization, the scale of panel C was increased by a factor 2 with respect to the other panels. In this view, the defect appears as a +1/2 trefoil. In both panels B and C, the director field around the disclination line is highlighted with black curves. The dimensions of the region in panel A are $300 \times 300 \times 100\ \mu m^3$.

bioRxiv preprint doi: https://doi.org/10.1101/2025.04.14.648711; this version posted May 10, 2025. The copyright holder for this preprint (which was not certified by peer review) is the author/funder, who has granted bioRxiv a license to display the preprint in perpetuity. It is made available under aCC-BY 4.0 International license.

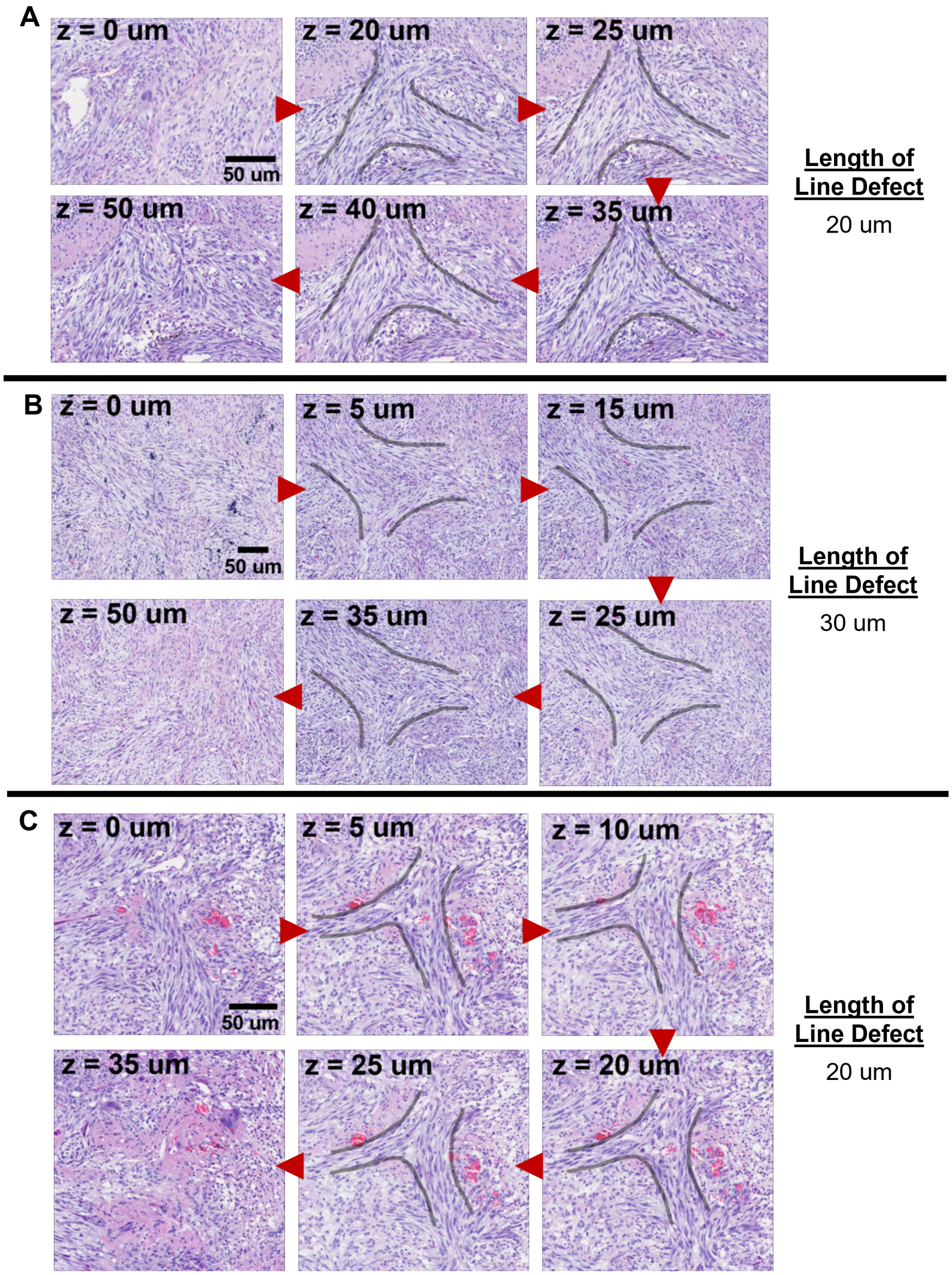

Figure S11: **Trefoil Topological Defect Lines in SB NPA Tumor** #2 **(A-C)** Three examples of H&E stained serial sections of Sleeping Beauty NPA #2 tumor highlighting trefoil topological defects over several slices. Each image shows the same region within the tumor at different z depth, as indicated in the top left corner. Red arrows show progression into the z-axis of the tumor. Gray curves outline trefoils. The length of the defect sequences is indicated on the right. Scale bar = 50 μm.

bioRxiv preprint doi: https://doi.org/10.1101/2025.04.14.648711; this version posted May 10, 2025. The copyright holder for this preprint (which was not certified by peer review) is the author/funder, who has granted bioRxiv a license to display the preprint in perpetuity. It is made available under aCC-BY 4.0 International license.

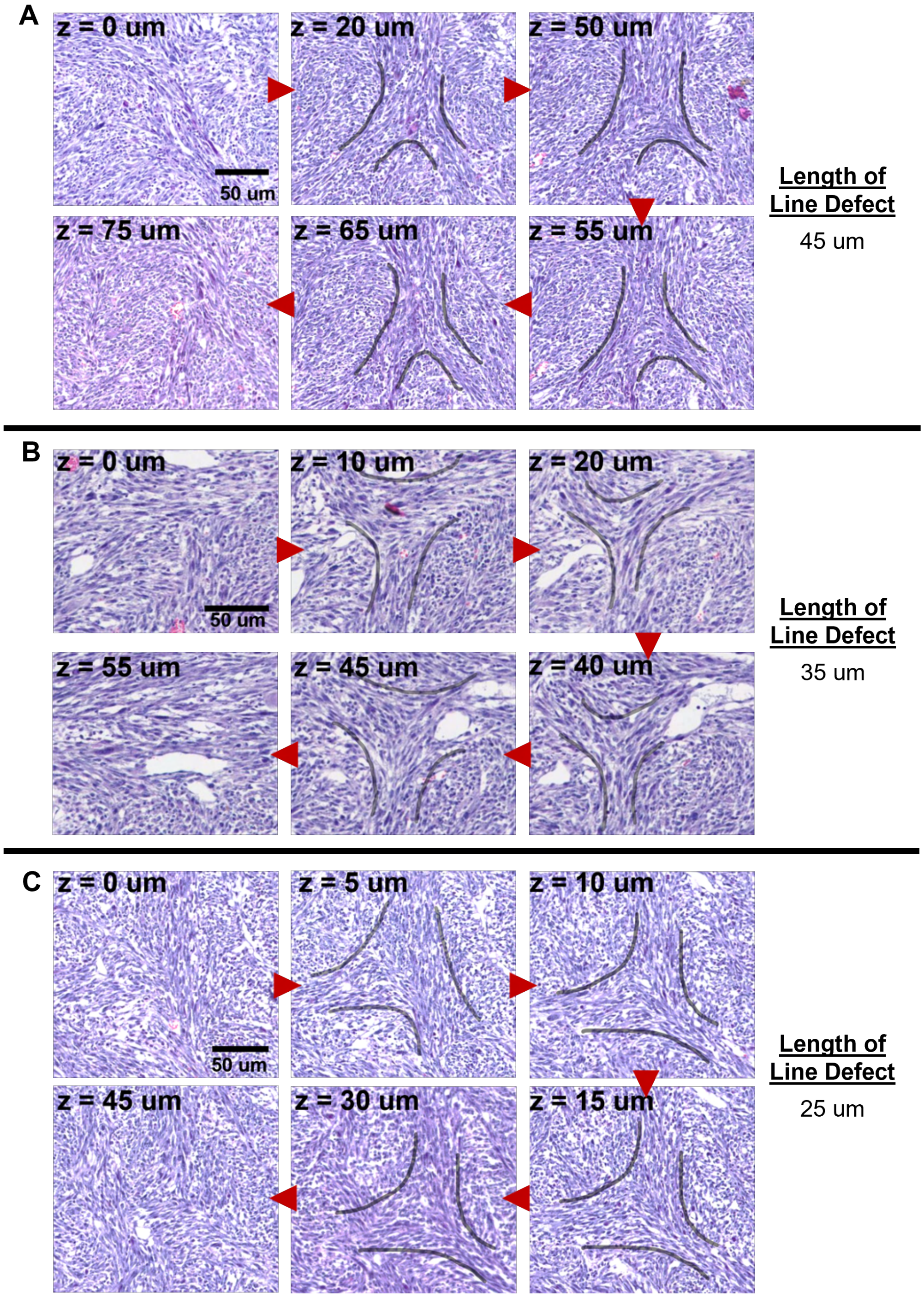

Figure S12: **Trefoil Topological Defect Lines in SB NPD Tumor** #1 **(A-C)** Three examples of H&E stained serial sections of intracranial NPD #1 tumor highlighting trefoil topological defects over several slices. Each image shows the same region within the tumor at different z depth, as indicated in the top left corner. Red arrows show progression into the z-axis of the tumor. Gray curves outline trefoils. The length of the defect sequences is indicated on the right. Scale bar = 50 μm.

bioRxiv preprint doi: https://doi.org/10.1101/2025.04.14.648711; this version posted May 10, 2025. The copyright holder for this preprint (which was not certified by peer review) is the author/funder, who has granted bioRxiv a license to display the preprint in perpetuity. It is made available under aCC-BY 4.0 International license.

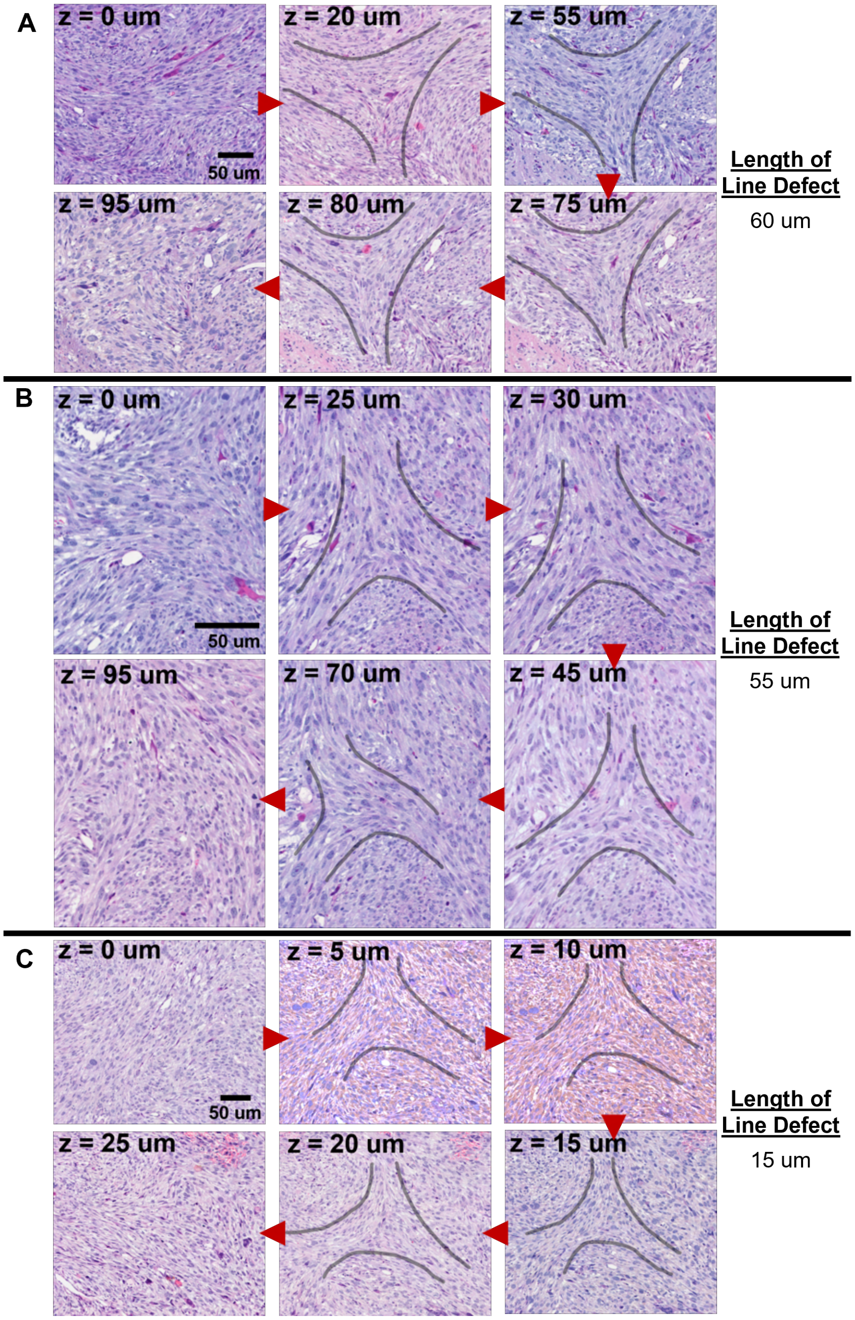

Figure S13: **Trefoil Topological Defect Lines in SB NPA Tumor** #1 **(A-C)** Three examples of H&E stained serial sections of Sleeping Beauty NPA #1 tumor highlighting trefoil topological defects over several slices. Each image shows the same region within the tumor at different z depth, as indicated in the top left corner. Red arrows show progression into the z-axis of the tumor. Gray curves outline trefoils. The length of the defect sequences is indicated on the right. Scale bar = 50 μm.

bioRxiv preprint doi: https://doi.org/10.1101/2025.04.14.648711; this version posted May 10, 2025. The copyright holder for this preprint (which was not certified by peer review) is the author/funder, who has granted bioRxiv a license to display the preprint in perpetuity. It is made available under aCC-BY 4.0 International license.

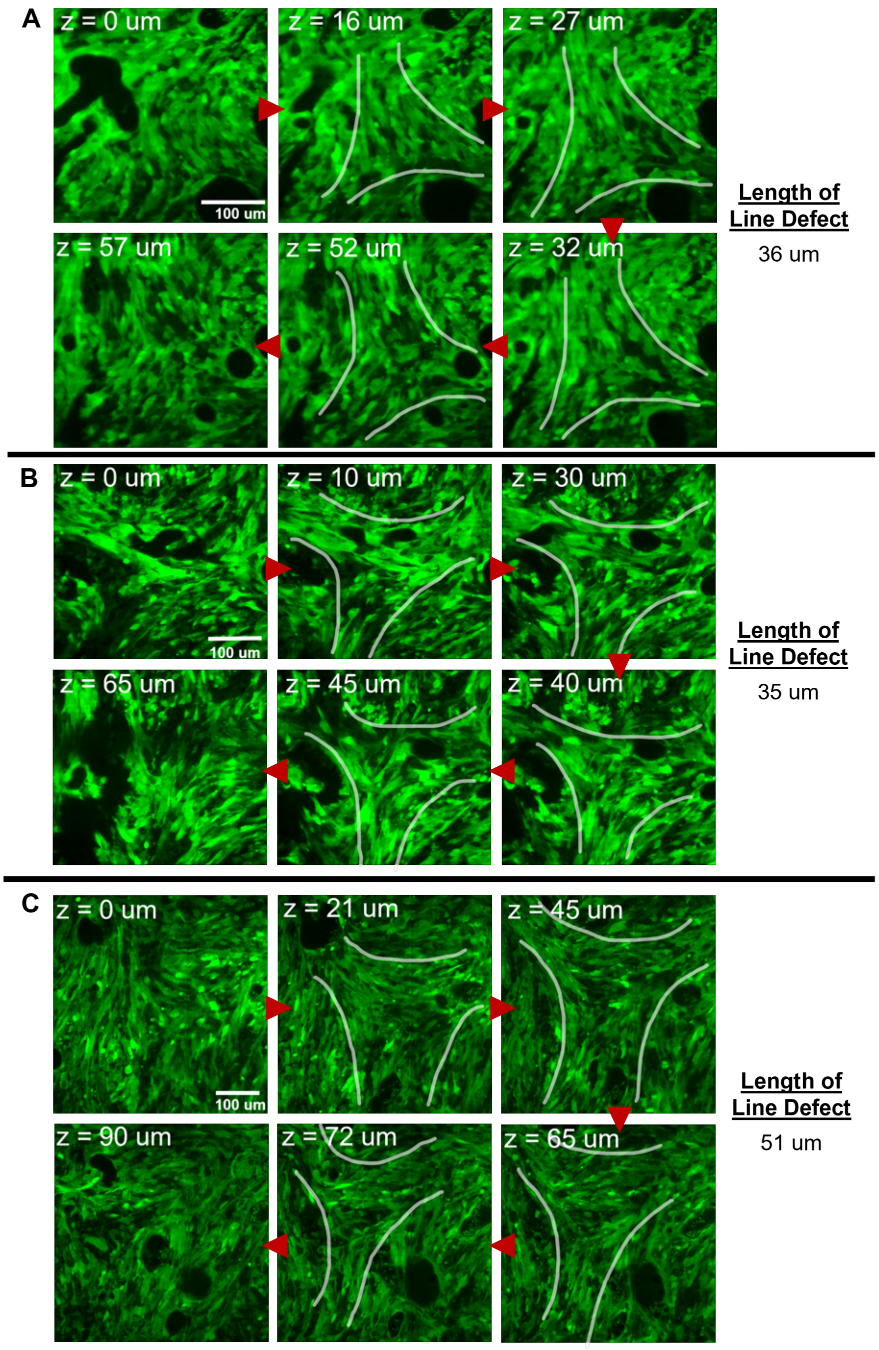

Figure S14: **Trefoil Topological Defect Lines in Cleared Intracranial NPD Tumor (A-C)** Three examples of LSM imaged cleared intracranial NPD tumors (n = 3) highlighting trefoil topological defects over several slices. Each image shows the same region within the tumor at different z depth, as indicated in the top left corner. Red arrows show progression into the z-axis of the tumor. Gray curves outline trefoils. The length of the defect sequences is indicated on the right. Scale bar = 100 μm.

bioRxiv preprint doi: https://doi.org/10.1101/2025.04.14.648711; this version posted May 10, 2025. The copyright holder for this preprint (which was not certified by peer review) is the author/funder, who has granted bioRxiv a license to display the preprint in perpetuity. It is made available under aCC-BY 4.0 International license.

Figure S15: Comet Topological Defect Lines in Cleared Intracranial NPD Tumor

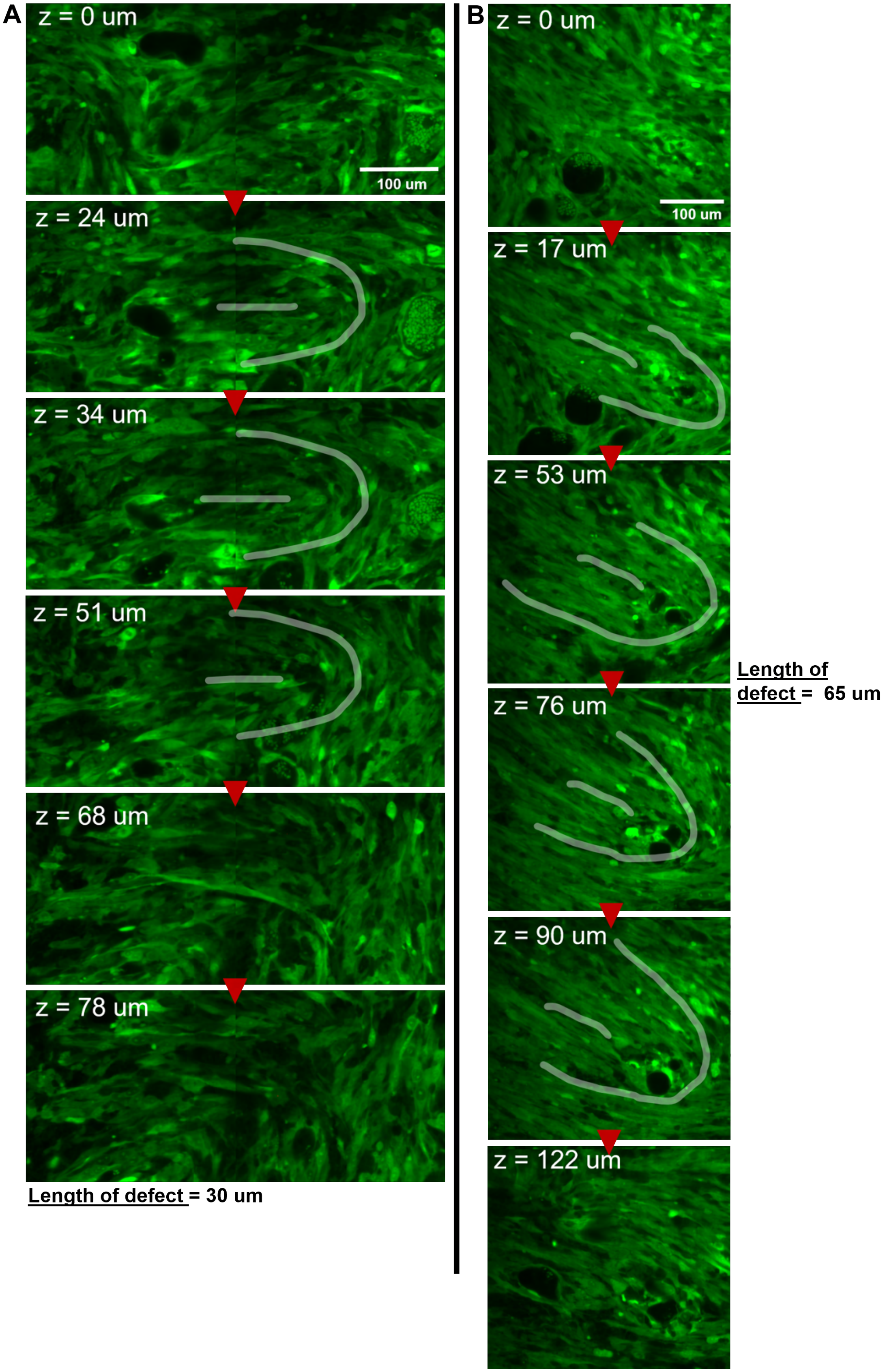

bioRxiv preprint doi: https://doi.org/10.1101/2025.04.14.648711; this version posted May 10, 2025. The copyright holder for this preprint (which was not certified by peer review) is the author/funder, who has granted bioRxiv a license to display the preprint in perpetuity. It is made available under aCC-BY 4.0 International license.

Figure S15: **Comet Topological Defect Lines in Cleared Intracranial NPD Tumor (A-B)** Two examples of LSM imaged cleared intracranial NPD tumors highlighting comet topological defects over several slices. Each image shows the same region within the tumor at different z depth, as indicated in the top left corner. Red arrows show progression into the z-axis of the tumor. Gray curves outline comets. The length of the defect sequences is indicated. Scale bar = 100 μm.

bioRxiv preprint doi: https://doi.org/10.1101/2025.04.14.648711; this version posted May 10, 2025. The copyright holder for this preprint (which was not certified by peer review) is the author/funder, who has granted bioRxiv a license to display the preprint in perpetuity. It is made available under aCC-BY 4.0 International license.

Figure S16: Dynamics of nematic order and topological defects in glioma cell cultures

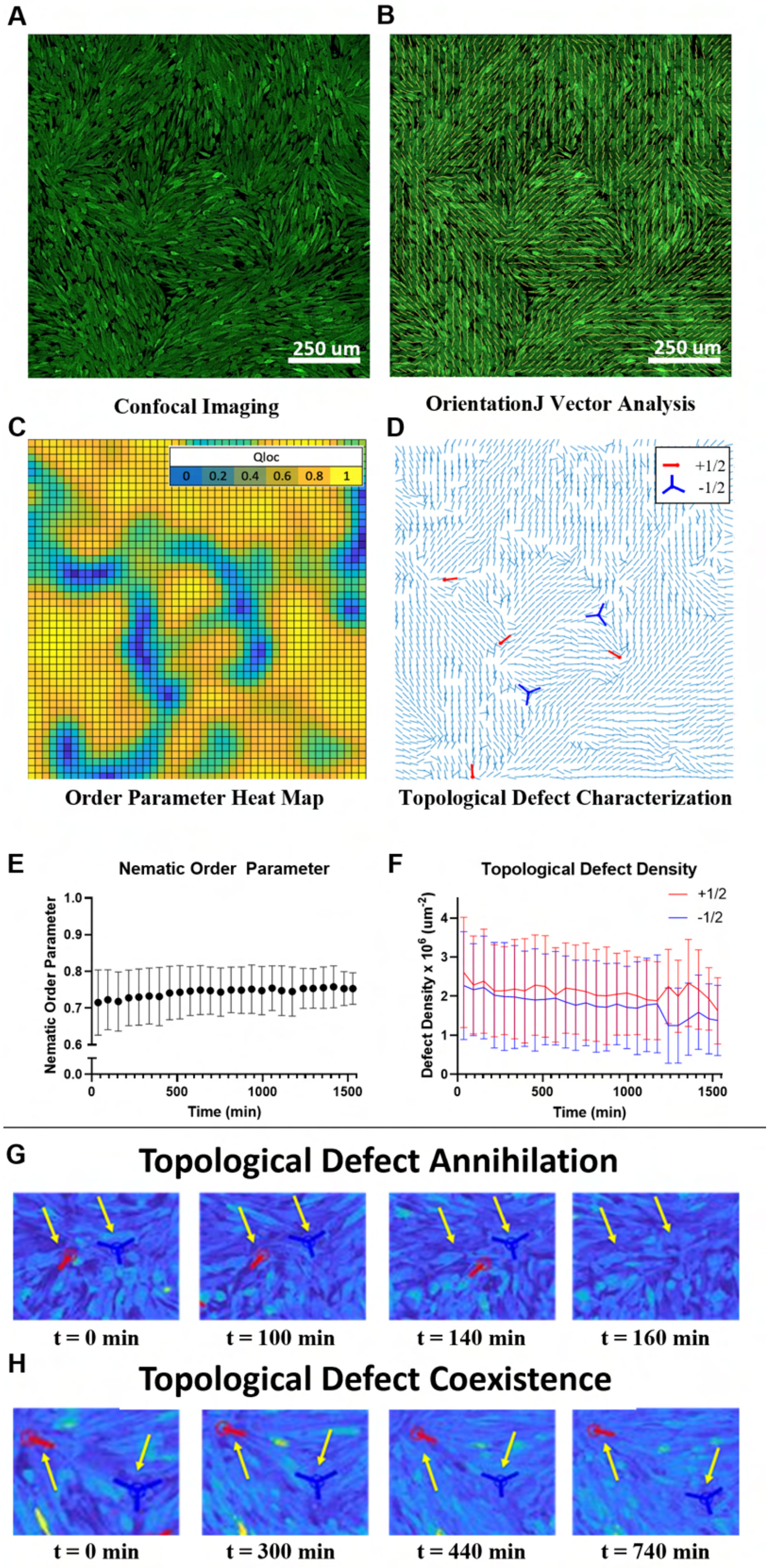

bioRxiv preprint doi: https://doi.org/10.1101/2025.04.14.648711; this version posted May 10, 2025. The copyright holder for this preprint (which was not certified by peer review) is the author/funder, who has granted bioRxiv a license to display the preprint in perpetuity. It is made available under aCC-BY 4.0 International license.

Figure S16: **Dynamics of nematic order and topological defects in glioma cell cultures (A)** NPA glioma cells (cytosol tagged with green fluorescent protein (GFP)) imaged with confocal microscopy. **(B)** 2D director field (yellow dashes) for the image in panel A. **(C)** Heatmap of local order parameter, $Qloc$ with coarse-graining length equal to 25 μm for the same image in panel A. $Qloc = 0$ (dark blue) represents random alignment and $Qloc = 1$ (bright yellow) represents perfect nematic alignment. **(D)** Identification of topological defects with $-1/2$ defects (trefoils) in dark blue and $+1/2$ (comets) in red for the same image in panel A. Light blue dashes show the 2D director field. **(E)** Time evolution of the averaged $Qloc$. **(F)** Time evolution of the averaged topological defect density for $-1/2$(dark blue) and $+1/2$ (red) defects. **(G)** An example of the annihilation of a pair of topological defects with opposite charge. **(H)** An example of the extended coexistence of a pair of topological defects with opposite charge. For A,B: scale bars = 250 μm. For E,F: $n = 6$ movies, $N = 40$ imaged positions; error bars correspond to SD; and time $t = 0$ min corresponds to one day post cell seeding. For G, H: scale bars = 10 μm and $t = 0$ min was set arbitrarily.

bioRxiv preprint doi: https://doi.org/10.1101/2025.04.14.648711; this version posted May 10, 2025. The copyright holder for this preprint (which was not certified by peer review) is the author/funder, who has granted bioRxiv a license to display the preprint in perpetuity. It is made available under aCC-BY 4.0 International license.

Figure S17: Analysis of the motion of $+1/2$ topological defects

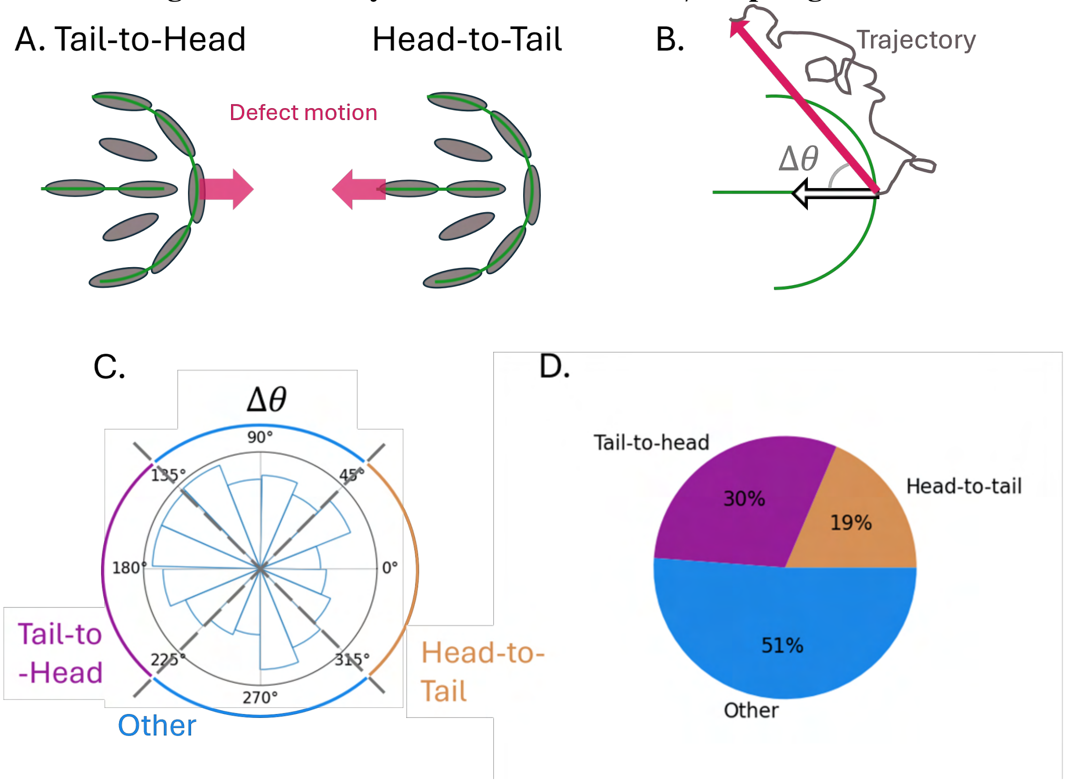

Figure S17: **Analysis of the motion of $+1/2$ topological defects (A)** Two schematics of $+1/2$ topological defects (director field outline in green curves). Cells are colored in gray. The magenta arrows indicate the direction of defect motion for a tail-to-head moving defects (left panel) or a head-to-tail moving defect (right panel). **(B)** Schematic of a $+1/2$ topological defect with director field outlined in green. The gray curve represents the defect trajectory and the magenta arrow the displacement vector from the initial time point to the last time point. The empty arrow is the polarization $\mathbf{p}$ of the topological defect. In practice, the polarization vector was computed as the mean polarization vector over the trajectory. The angular difference between the mean polarization and the displacement vector is $\Delta\theta$. **(C)** Polar histogram of $\Delta\theta$ for $N = 172$ independent $+1/2$ topological defects. The trajectories are classified as 'head-to-tail', if $-45° < \Delta\theta < 45°$ (orange range), 'tail-to-head', if $225° < \Delta\theta < 135°$ (purple range), and 'other' in any other case (blue range). **(D)** Fraction of $+1/2$ topological defect with a motion head-to-tail, tail-to-head, or other.

bioRxiv preprint doi: https://doi.org/10.1101/2025.04.14.648711; this version posted May 10, 2025. The copyright holder for this preprint (which was not certified by peer review) is the author/funder, who has granted bioRxiv a license to display the preprint in perpetuity. It is made available under aCC-BY 4.0 International license.

Caption for Movie S1: (LSM Z-Stack) Raw light-sheet microscopy z-stack image of GFP-tagged (green) glioma NPD in vivo tumor. Z-planes are 1 μm apart. The cell orientations form a trefoil-like shape in 2D which propagates in the z-direction. The -1/2 defect is analyzed in detail in Fig. 3 and fig. S10.

Caption for Movie S2: (Omega Vector Rotations) Rotating view of nematic defects in the cleared intracranial NPD mouse glioma region shown in Fig. 3, with analysis of defect winding geometry. Transparent purple surfaces are isosurfaces of nematic order bounding low-order regions, including topological defects, as in Fig. 3. Pink surfaces indicate blood vessels. At a sampled subset of points on the low-order surfaces, the nematic director field is plotted as teal cylinders at nearby points located on a small circuit, which encircles the associated point on the defect provided the surface is tube-like there. Double-headed arrows (shaded green when twist-type to yellow when wedge-type) show the rotation vector, Ω (with sign ambiguity) at those defect points, computed from the normal to the plane of rotation of the director on the circuit.

Caption for Movie S3: (X-Y View of Fig 3) Animation of changing z-slice coordinate in a three-dimensional view of the nematic order and defects in the cleared intracranial NPD mouse glioma region shown in Fig. 3. On each slice of constant z, gray-scale LSM intensity is plotted along with the inferred nematic director field (teal cylinders). Purple surfaces are isosurfaces of nematic order bounding low-order regions, including topological defects, as in Fig. 3. Pink surfaces indicate blood vessels.

Caption for Movie S4: (X-Z View of Fig 3) Animation of changing y-slice coordinate in a three-dimensional view of the nematic order and defects in the cleared intracranial NPD mouse glioma region shown in Fig. 3. On each slice of constant y, gray-scale LSM intensity is plotted along with the inferred nematic director field (teal cylinders). Purple surfaces are isosurfaces of nematic order bounding low-order regions, including topological defects, as in Fig. 3. Pink surfaces indicate blood vessels.

Caption for Movie S5: (05-03-23-Pos3-GFP) 20x in vitro confocal movie showing formation of trefoils and comets in GFP-tagged (green) glioma NPA adherent cells over 70 hours. Movie

bioRxiv preprint doi: https://doi.org/10.1101/2025.04.14.648711; this version posted May 10, 2025. The copyright holder for this preprint (which was not certified by peer review) is the author/funder, who has granted bioRxiv a license to display the preprint in perpetuity. It is made available under aCC-BY 4.0 International license.

analyzed in fig. S16, A-D.

Caption for Movie S6: (07-12-22-Pos2-GFP) 20x in vitro confocal movie showing formation of trefoils and comets in GFP-tagged (green) glioma NPA adherent cells over 25 hours.

Caption for Movie S7: (05-19-23-Pos4-Casp3) 20x in vitro confocal movie showing formation of trefoils and comets in GFP-tagged (green) glioma NPA adherent cells over 70 hours with apoptosis labeled in red.

Caption for Movie S8: (05-19-23-Pos9-Casp3) 20x in vitro confocal movie showing formation of trefoils and comets in GFP-tagged (green) glioma NPA adherent cells over 70 hours with apoptosis labeled in red.
